# A host cell long noncoding RNA NR_033736 regulates type I interferon-mediated gene transcription and modulates intestinal epithelial anti-*Cryptosporidium* defense

**DOI:** 10.1371/journal.ppat.1009241

**Published:** 2021-01-22

**Authors:** Juan Li, Kehua Jin, Min Li, Nicholas W. Mathy, Ai-Yu Gong, Silu Deng, Gislaine A. Martins, Mingfei Sun, Juliane K. Strauss-Soukup, Xian-Ming Chen

**Affiliations:** 1 Institute of Animal Health, Guangdong Academy of Agricultural Sciences, Guangzhou, Guangdong, China; 2 Department of Medical Microbiology and Immunology, Creighton University School of Medicine, Omaha, NE, United States of America; 3 Department of Biochemistry, Hubei University of Science and Technology, Xianning, Hubei, China; 4 Department of Medicine and Biomedical Sciences, Research Division of Immunology Cedars-Sinai Medical Center, David Geffen School of Medicine, University of California, Los Angeles, Los Angeles, CA, United States of America; 5 Department of Chemistry, Creighton University College of Arts and Sciences, Omaha, NE, United States of America; University of Wisconsin Medical School, UNITED STATES

## Abstract

The gastrointestinal epithelium guides the immune system to differentiate between commensal and pathogenic microbiota, which relies on intimate links with the type I IFN signal pathway. Epithelial cells along the epithelium provide the front line of host defense against pathogen infection in the gastrointestinal tract. Increasing evidence supports the regulatory potential of long noncoding RNAs (lncRNAs) in immune defense but their role in regulating intestinal epithelial antimicrobial responses is still unclear. *Cryptosporidium*, a protozoan parasite that infects intestinal epithelial cells, is an important opportunistic pathogen in AIDS patients and a common cause of diarrhea in young children in developing countries. Recent advances in *Cryptosporidium* research have revealed a strong type I IFN response in infected intestinal epithelial cells. We previously identified a panel of host cell lncRNAs that are upregulated in murine intestinal epithelial cells following microbial challenge. One of these lncRNAs, NR_033736, is upregulated in intestinal epithelial cells following *Cryptosporidium* infection and displays a significant suppressive effect on type I IFN-controlled gene transcription in infected host cells. NR_033736 can be assembled into the ISGF3 complex and suppresses type I IFN-mediated gene transcription. Interestingly, upregulation of NR_033736 itself is triggered by the type I IFN signaling. Moreover, NR_033736 modulates epithelial anti-*Cryptosporidium* defense. Our data suggest that upregulation of NR_033736 provides negative feedback regulation of type I IFN signaling through suppression of type I IFN-controlled gene transcription, and consequently, contributing to fine-tuning of epithelial innate defense against microbial infection.

## Author summary

Intestinal epithelial cells are an important component of gastrointestinal mucosal immunity. They generate various types of barriers to protect the intestinal mucosa from commensal microbes or invading pathogenic microorganisms. Upon microbial infection, intestinal epithelial cells quickly initiate a series of innate immune reactions, including production of antimicrobial molecules and release of inflammatory chemokines and cytokines. These chemokines and cytokines of epithelial cell origin may mobilize and activate immune effector cells to the infection sites. One family of these cytokines are type I IFNs (e.g., IFN-α/β), which not only activate other immune effector cells at gastrointestinal mucosa but also stimulate epithelial function through ligation of receptors expressed on intestinal epithelial cells themselves in an autocrine manner. *Cryptosporidium* is an important opportunistic pathogen in AIDS patients and a common cause of diarrhea in young children in developing countries. Recent advances in *Cryptosporidium* research have revealed a strong type I IFN response in infected intestinal epithelium. In this study, we provide data demonstrating an important role for long noncoding RNAs (lncRNAs) in regulating type I IFN signaling in intestinal epithelial cells in response to *Cryptosporidium* infection. LncRNAs are recently identified long non-coding transcripts (>200 nt) that are not translated into protein. We found that upregulation of one host cell lncRNA, NR_033736, displays a significant suppressive effect on type I IFN-controlled gene transcription in intestinal epithelial cells following infection, contributing to the fine-tuning of epithelial anti-*Cryptosporidium* defense. Our data suggest a new arm of feedback regulation for type I IFN signaling through lncRNA-mediated gene transcription, contributing to fine-tuning of epithelial innate defense against microbial infection in the gastrointestinal tract.

## Introduction

Long non-coding RNAs (lncRNAs) are recently identified long non-coding transcripts (>200 nt) that are not translated into protein [1]. The lncRNA transcriptome exhibits both ubiquitously expressed and tissue-specific features [2]. A total 27,919 long ncRNAs have been identified in various human sources using the Functional Annotation of the Mouse/Mammalian Genome 5 (FANTOM5) approach [3]. Accumulating evidence suggests that the majority of these lncRNAs are likely to be functional [4, 5]. In most cases, lncRNAs function through specific interactions with other cellular factors, including proteins, DNA, and other RNA molecules [1, 6]. Some lncRNAs are induced in innate immune cells and are likely to play key roles in the regulation of innate defense [7]. For example, lncRNA-Cox2, one of the most highly induced lncRNAs in macrophages, has been shown to mediate both the activation and repression of distinct classes of immune genes [8]. In our previous studies, we demonstrated that lncRNA-Cox2 and lncRNA-Tnfaip3 regulate inflammatory gene transcription in intestinal epithelial cells through modulating ATP-dependent chromatin remodeling [9–11].

Interferons (IFNs) are broadly expressed cytokines that drive innate immunity, responding to pathogenic attack or injury with both pro- and anti-inflammatory responses. The IFN family is usually classified into three main types: type I (e.g., IFN-α and IFN-β), type II (IFN-γ), and type III (IFN-λ family) [12]. Type I IFNs can be produced by almost every cell type, including intestinal epithelial cells [13]. The gastrointestinal epithelium guides the immune system to differentiate between commensal and pathogenic microbiota, which relies on intimate links with the type I IFN signal pathway [14]. The current paradigm depicts a type I IFN-induced anti-proliferative state in the intestinal epithelium enabling cell differentiation, cell maturation, and proper intestinal barrier function, strongly supporting its role in maintaining baseline immune activity and clearance of damaged epithelia or pathogens [15]. Nevertheless, while type I IFNs have long been known for their protective role in viral infections, they can either protect or exacerbate bacterial infections and contribute to immune homeostasis in the intestine through both immune activating and suppressive signals [15]. Molecular mechanisms regulating type I IFN signal pathway in gastrointestinal epithelial cells in response to various pathogens are not fully understood.

*Cryptosporidium spp*, a coccidian parasite and an NIAID Category B priority pathogen, infects the gastrointestinal epithelium, causing a self-limited disease in immunocompetent individuals but a life-threatening diarrheal disease in AIDS patients [16, 17]. After rotavirus, *Cryptosporidium* is the most common pathogen responsible for moderate-to-severe diarrhea in children younger than 2 years in developing regions [18]. The majority of human cryptosporidial infections are caused by two species: *C*. *parvum* and *C*. *hominis* [16]. *C*. *parvum* attaches to the apical membrane surface of intestinal epithelial cells (mainly enterocytes) and forms an intracellular but extracytoplasmic vacuole in which the organism remains [19]. Thus, *C*. *parvum* is classified as a “minimally invasive” mucosal pathogen [19] and innate epithelial defense is critical to the host’s defense against *C*. *parvum* infection [20, 21]. Recent advances in *Cryptosporidium* research have revealed a strong type I IFN response in infected intestinal epithelium [22].

Using an *in vitro* infection model of intestinal epithelial cells by *C*. *parvum*, we recently identified a panel of host cell lncRNAs that are upregulated following *C*. *parvum* infection [23]. In this study, we present data demonstrating that one of these upregulated lncRNAs in infected cells, NR_033736 [24], displayed a significant suppressive effect on type I IFN-controlled gene transcription in intestinal epithelial cells in response to cryptosporidial infection. Interestingly, upregulation of NR_033736 itself was controlled by the type I IFN signaling. Manipulation of NR_033736 expression in intestinal epithelial cells revealed significant impact on epithelial anti-*C*. *parvum* defense. Our data support that NR_033736 provides negative feedback regulation of type I IFN signaling through suppression of type I IFN-controlled gene transcription, and consequently, contributing to fine regulation of epithelial innate defense against cryptosporidial infection.

## Results

### Upregulation of NR_033736 expression and activation of type I IFN signaling in intestinal epithelium following *Cryptosporidium* infection

**Fig 1:**
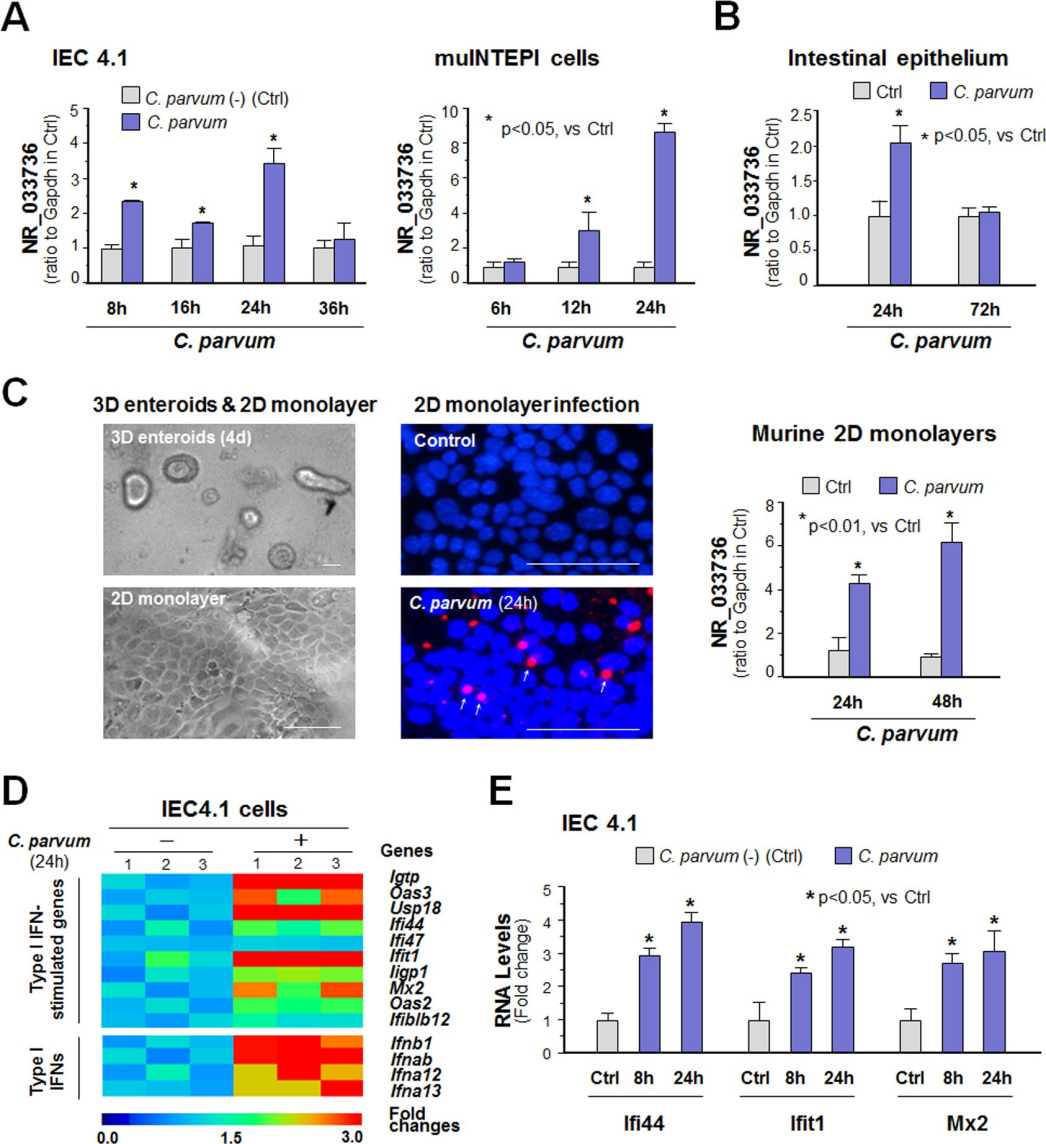
Upregulation of NR_033736 and activation of type I IFN signaling in intestinal epithelium following *Cryptosporidium* infection. (A) Upregulation of NR_033736 in cultured murine intestinal epithelial cells (IEC4.1 and muINTEPI cells) following *C*. *parvum* infection. IEC4.1 and muINTEPI cells were exposed to *C*. *parvum* infection for 6–36 h and expression level of NR_033736 was validated by using qRT-PCR. (B) Upregulation of NR_033736 in murine intestinal epithelium following *C*. *parvum* infection *in vivo*. Neonates of mice at 5 days of age received *C*. *parvum* administration by oral and intestinal ileum epithelium were isolated after infection for 24–72 h. Expression level of NR_033736 was quantified by using qRT-PCR. (C) Upregulation of NR_033736 in 2D murine intestinal epithelial monolayers following *C*. *parvum* infection *ex vivo*. The crypt units of small intestinal epithelium from neonates of 5 days old were isolated and cultured into 2D monolayers followed by exposure to *C*. *parvum* infection for 24–48 h, as shown by phase and immunofluorescence microscopy. NR_033736 expression was validated by using qRT-PCR. (D) Upregulation of the selected type I IFN-controlled genes and type I IFN genes in IEC4.1 cells following *C*. *parvum* infection for 8 h. The expression levels of the genes were quantified and presented as a heatmap representing fold changes in infected cells compared with that in non-infected control. (E) Expression levels of selected type I IFN-controlled genes (i.e., *Ifi44*, *Ifit1*, and *Mx2*) in IEC4.1 cells following *C*. *parvum* infection for 8h and 24 h. Data represent three independent experiments. *P < .05 vs cells of non-infected control (Ctrl).

We previously performed a genome-wide transcriptome analysis of *C*. *parvum*-infected IEC4.1 cells, transformed but non-tumorigenic intestinal epithelial cells from neonatal mice (5–7 days old) [25] and received from Dr. Pingchang Yang (McMaster University, Hamilton, Canada). Following infection for 24h, IEC4.1 cells demonstrated a significant elevated expression profile of many host cell lncRNAs [23]. One of the top upregulated lncRNAs is NR_033736 (which was 2.00 ± 0.12 fold increase in infected cells vs non-infected control, p<0.05). This lncRNA is transcribed from a gene locus (*E330020D12Rik*) localized by the *Dhx9* and *Shcbp1l* genes [24]. Using quantitative real-time PCR (qRT-PCR), we confirmed the upregulation of NR_033736 in IEC4.1 cells and in the murine intestinal epithelial cell line (muINTEPI cells) [26] following *C*. *parvum* infection (1.80 to 3.31 fold increase at 8–24 h post-infection and 2.42 to 8.45 fold increase at 12–24 h post-infection, p<0.05, respectively, Fig 1A). Given the higher susceptibility of neonatal mice to *C*. *parvum* infection, we focused hereby on IEC4.1 cells for our *in vitro* infection analysis. Upregulation of NR_033736 was found in isolated intestinal epithelium from neonatal mice of intestinal cryptosporidiosis through oral administration of the parasite [27, 28] (2.01 ± 0.12 fold increase at 24 h post-infection, p<0.05, Fig 1B). Using an *ex vivo* infection model employing 2D enteroid monolayers from neonatal mouse ileum [29–31] (Fig 1C), we also detected upregulation of NR_033736 in intestinal epithelial monolayers following *C*. *parvum* infection (4.03 ± 0.10 fold increase at 24–48 h post-infection, p<0.001, Fig 1C). Consistent with previous studies showing a strong type I IFN response in infected intestinal epithelium [22], we detected upregulation of type I IFN-controlled genes and several type I IFNs in IEC4.1 cells following *C*. *parvum* infection (Fig 1D and 1E and S1 Table). Type I IFN-controlled genes include interferon gamma induced GTPase (*Igtp*), 2’-5’-oligoadenylate synthetase 3 (*Oas3*), ubiquitin specific peptidase 18 (*Usp18*), interferon induced protein 44 (*Ifi44*), interferon induced protein with tetratricopeptide repeats 1 (*Ifit1*), interferon inducible GTPase 1 (*Iigp1*), and MX dynamin-like GTPase 2 (*Mx2*) (a full list is available in S1 Table). Type I IFN genes include interferon beta 1 (*Ifnb1*), interferon alpha B (*Ifnab*), *Ifna12*, and *Ifna13*.

### Upregulation of NR_033736 is triggered by type I IFN signaling in intestinal epithelial cells and macrophages

**Fig 2:**
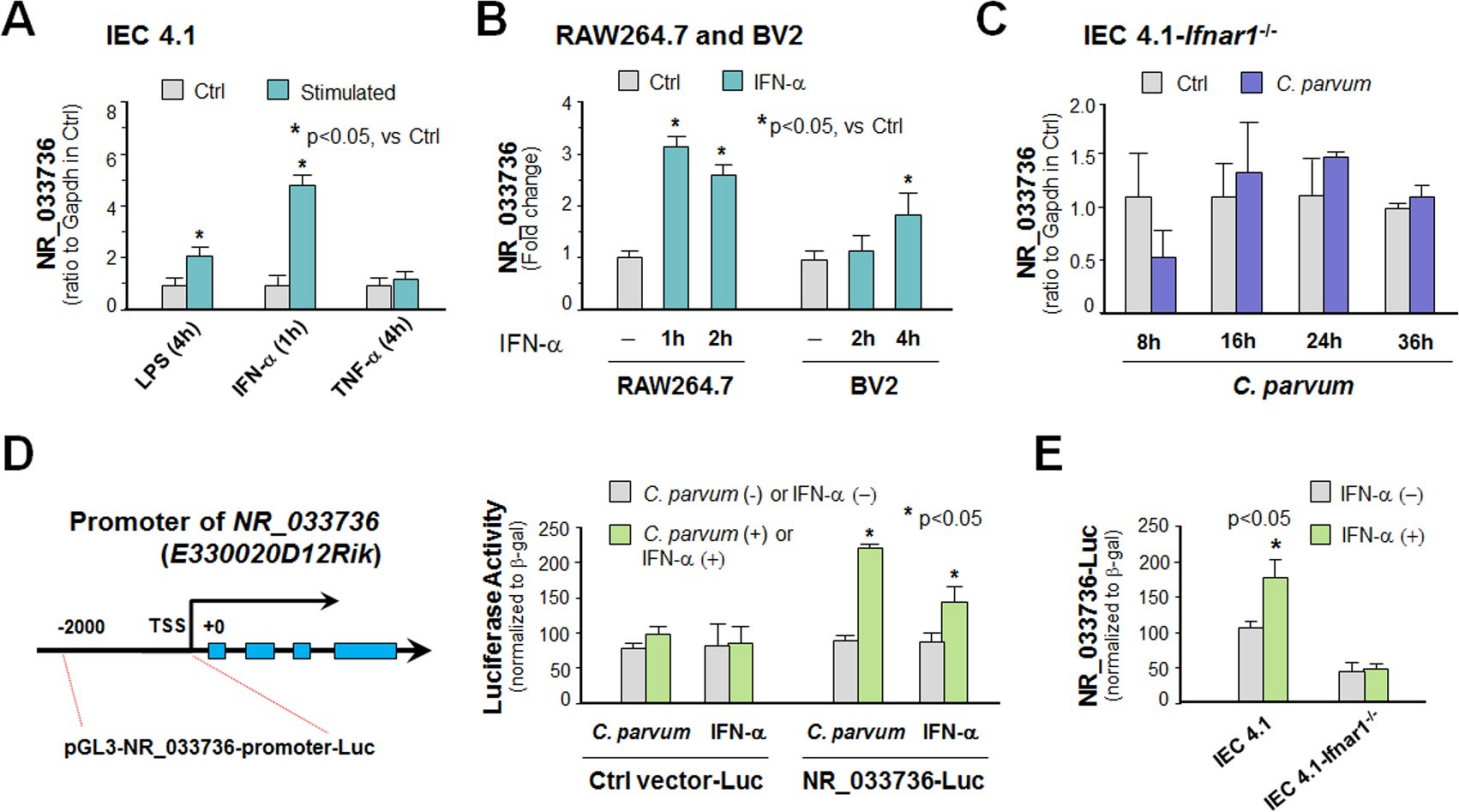
Upregulation of NR_033736 is triggered by type I IFN signaling in intestinal epithelial cells and macrophages. (A) Upregulation of NR_033736 in IEC4.1 cells following stimulation with LPS, IFN-α and TNF-α. Cells were treated with LPS, IFN-α and TNF-α for 2 h and expression level of NR_033736 was validated by using qRT-PCR. (B) Upregulation of NR_033736 in cultured murine macrophages (RAW287.4 cells) and microglia (BV2 cells) following IFN-α stimulation. Cells were treated with IFN-α for 2 h and expression level of NR_033736 was quantified by using qRT-PCR. (C) Upregulation of NR_033736 induced by *C*. *parvum* infection was not observed in IEC4.1 cells with deficient in *Ifnar1*. Stable IEC4.1 cells were exposed to *C*. *parvum* infection for 8–36 h and expression level of NR_033736 was quantified. (D) *C*. *parvum* infection and IFN-α stimulation trigger the luciferase activity associated with the NR_033736 promoter in IEC4.1 cells. The 2-kb upstream of the transcription start site of NR_033736 was cloned and inserted into the pGL3-Basic luciferase reporter construct. Cells were transfected with the generated reporter constructs and then exposed to *C*. *parvum* infection (for 8 h) or IFN-α stimulation (for 4 h). Infection and IFN-α stimulation triggered the luciferase activity associated with the NR_033736 promoter in IEC4.1 cells. (E) Luciferase activity associated with the NR_033736 promoter in IEC4.1 cells with deficient in *Ifnar1* in response to IFN-α stimulation. Cells were transfected with the generated reporter constructs and then treated with IFN-α for 4 h, followed by measurement of luciferase activity associated with the NR_033736 promoter. Data represent three independent experiments.

We then investigated what signaling pathways may control NR_033736 expression in intestinal epithelial cells following infection. Multiple intracellular signals are activated in host cells in response to *C*. *parvum* infection, including the NF-кB, type I IFN, and JNK/MAPK pathways [20, 32–34]. Upregulation of NR_033736 was detected in IEC4.1 cells stimulated with IFN-α (R&D, 50 IU/ml) for 1h, but in a much lower level in cells treated with lipopolysaccharide (LPS, 1 μg/ml) and no change after TNF-α stimulation (R&D, 10 ng/ml) (Fig 2A). Upregulation of NR_033736 and type I IFN-controlled genes upon IFN-α stimulation was also observed in cultured RAW264.7 macrophages and microglial BV2 cells (Figs 2B and S1). *C*. *parvum*-induced upregulation of NR_033736 was not observed in IEC4.1 cells deficient in *Ifnar1* (CRISPR/Cas9 stable knockout cell line, lacking Ifnar1 the receptor subunit for type I IFN signaling) [35] (Figs 2C and S2).

To further test the control of NR_033736 expression by type I IFNs, we cloned the putative -2kb promoter region of the *NR_033736* (*E330020D12Rik*) gene and inserted the sequence into the luciferase reporter vector (Fig 2D). *C*. *parvum* infection or IFN-α stimulation increased the luciferase activity in IEC4.1 cells transfected with the luciferase construct that encompassed the promoter region of the gene, but not in cells transfected with the empty vector control (Fig 2D) or in IEC4.1 cells deficient in *Ifnar1* (Fig 2E). These above data suggest that NR_033736 is a type I IFN-stimulated lncRNA and *C*. *parvum* infection triggers NR_033736 expression through activation of the type I IFN signaling pathway in intestinal epithelial cells.

### NR_033736 modulates type I IFN-controlled gene transcription in intestinal epithelial cells

**Fig 3:**
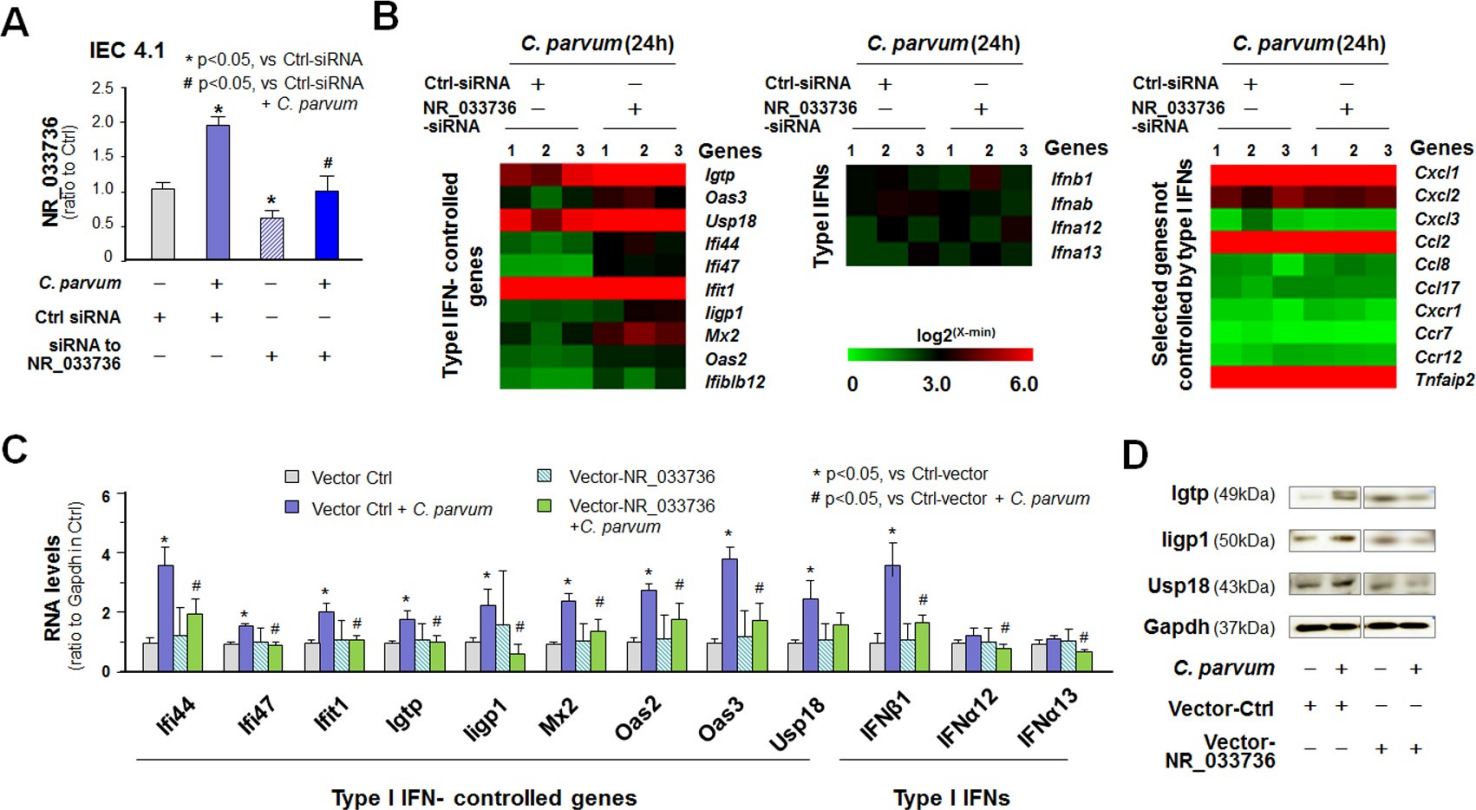
NR_033736 modulates type I IFN-controlled gene transcription in intestinal epithelial cells in response to *C*. *parvum* infection. (A) Knockdown of NR_033736 expression with the siRNA in IEC4.1 cells in response to *C*. *parvum* infection. Cells were treated with the siRNA to NR_033736 for 24 h and then exposed to *C*. *parvum* infection for 8 h. Expression level of NR_033736 was validated by using qRT-PCR. A non-specific scrambled siRNA was used as the siRNA control (Ctrl-siRNA). (B) Heatmaps representing upregulation of the top 10 type I IFN-controlled genes, as well as selected type I IFN genes and inflammatory genes not directly regulated by type I IFNs, in IEC4.1 cells treated with Ctrl-siRNA or siRNA-NR_033736 following *C*. *parvum* infection. IEC4.1 cells were treated with the Ctrl-siRNA or siRNA-NR_033736 for 24 h and then exposed to *C*. *parvum* infection for 24 h followed by genome-wide array analysis. (C) Overexpressing NR_033736 attenuated the expression of type I IFN-controlled genes in IEC4.1 cells following *C*. *parvum* infection. Cells were transfected with either the control vector (Ctrl-vector) or vector-NR_033736 for 24 h and then exposed to *C*. *parvum* infection for 24 h. Expression levels of type I IFN-controlled genes and selected type I IFN genes were measured. Data represent three independent experiments. (D) Effects of overexpressing NR_033736 on the expression of selected type I IFN-controlled genes, *Igtp*, *Iigp1*, and *Usp18*, at their protein levels in IEC4.1 cells following *C*. *parvum* infection. Cells were transfected with either the control vector (Ctrl-vector) or vector-NR_033736 for 24 h and then exposed to *C*. *parvum* infection for 24 h, followed by Western blot analysis. Gapdh was also blotted for control and representative blotting gels are shown.

A small interfering RNA (siRNA) was designed to knockdown NR_033736 and a scrambled non-specific siRNA was used as the control. While the control siRNA showed no effect on NR_033736 expression, the siRNA to NR_033736 significantly (p<0.05) decreased NR_033736 basal expression level in IEC4.1 cells and blocked its upregulation in *C*. *parvum*-infected cells to the basal expression level as in the control siRNA-treated non-infected cells (Fig 3A). We then measured the effects of NR_033736-siRNA knockdown on *C*. *parvum*-induced gene transcription in IEC4.1 cells by whole-transcriptome analysis. Silencing of NR_033736 revealed significant alterations in *C*. *parvum*-induced expression of host genes in IEC 4.1 cells and a full list of these genes is listed in S2 Table. All RNA-Seq data were deposited at GEO database (with the accession number: GSE145464; https://www.ncbi.nlm.nih.gov/geo). Interestingly, expression levels of type I IFN-controlled genes, including *Ifit1*, *Ifi44*, *Iigp1*, *Oas2*, and *Mx2*, were globally further elevated in *C*. *parvum*-infected cells treated with the NR_033736 siRNA (Fig 3B and S2 Table). In contrast, silencing of NR_033736 showed no obvious effects on *C*. *parvum*-induced expression of type I IFN genes and inflammatory genes that are not regulated by IFNs (Fig 3B). Complementarily, we generated a vector expressing the 926 nt sequence of NR_033736 (Vector-NR_033736) (S2 Fig). When IEC4.1 cells overexpressing NR_033736 through transfection of the Vector-NR_0333736 (S3 Fig) were exposed to *C*. *parvum* infection, a global suppression of type I IFN-controlled gene expression was confirmed (Fig 3C). Moreover, upregulation of selected type I IFN-controlled genes (*Usp18*, *Igtp* and *Iigp1*) at the protein level was further observed using Western blot in infected IEC4.1 cells (Fig 3D). Increase in *Usp18*, *Igtp* and *Iigp1* content was not observed in infected IEC4.1 cells overexpressing NR_033736 (Fig 3D). Suppression of type I IFN-controlled gene expression by overexpressing NR_033736 in infected cells is not due to increased RNA degradation, as their mRNA stability was not altered in IEC4.1 cells overexpressing NR_033736 (mRNA stability of representative gene *Ifit1* is shown in S4 Fig).

**Fig 4:**
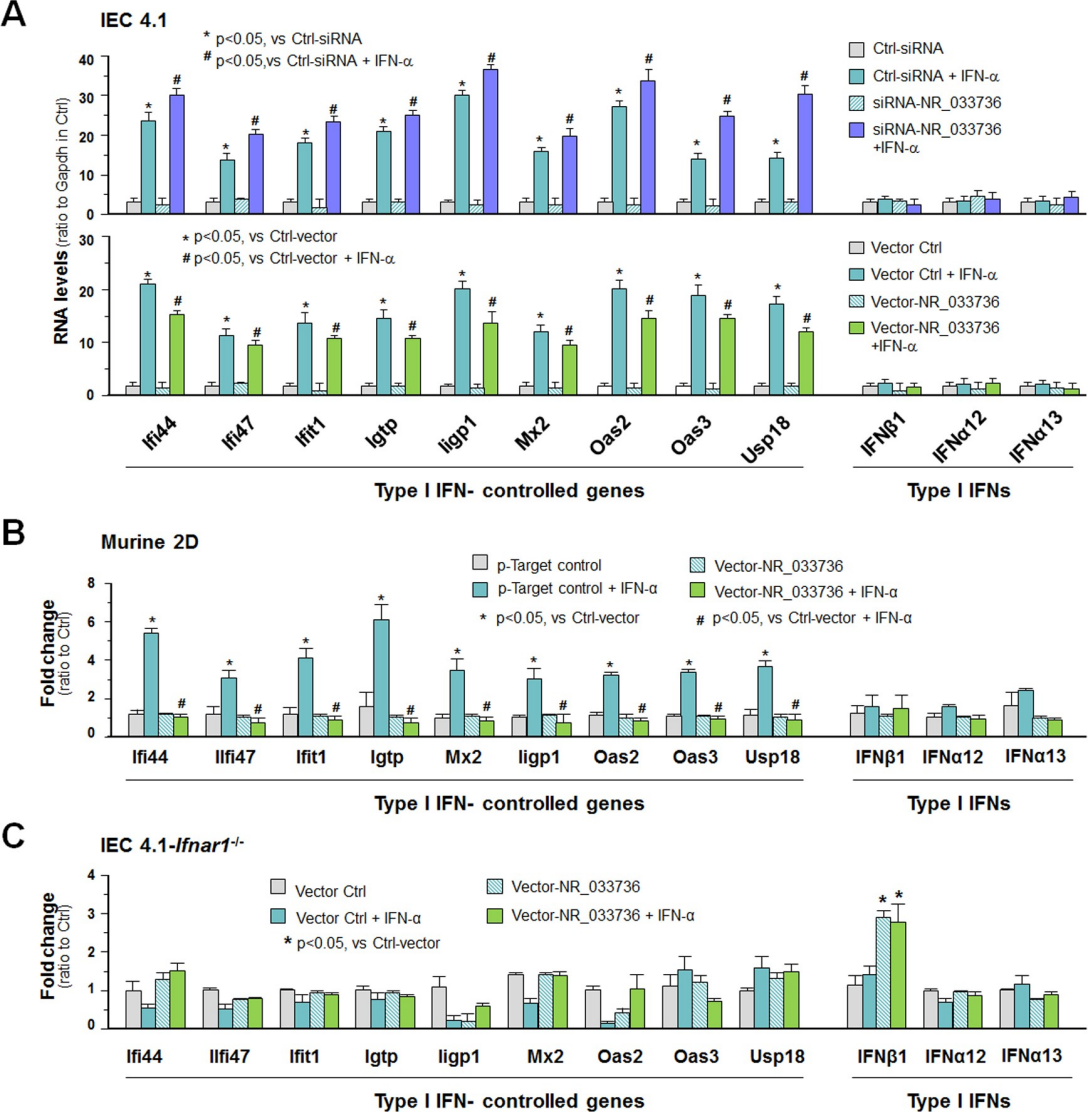
NR_033736 modulates type I IFN-mediated gene transcription in intestinal epithelial cells in response to IFN-α stimulation. (A) Effects of NR_033736 expression levels on IEC4.1 cell response to IFN-α stimulation. Cells were treated with the siRNA or transfected with the vector expression NR_033736 (Vector-NR_033736) for 24 h and then exposed to IFN-α stimulation for 2 h. Expression levels of type I IFN-controlled genes and selected type I IFN genes were validated by using qRT-PCR. Cells treated with the non-specific scrambled siRNA (Ctrl-siRNA) or the empty vector (Vector-Ctrl) were used for control. (B) Overexpressing NR_033736 attenuated the induction of type I IFN-controlled genes in murine intestinal epithelial 2D monolayers in response to IFN-α stimulation. The crypt units of small intestinal epithelium from neonates of 5 days old were isolated and cultured into 2D monolayers. Epithelial monolayers were then transfected with the Vector-NR_033736 for 24 h followed by exposure to IFN-α stimulation for 2 h. Expression levels of type I IFN-controlled genes and selected type I IFN genes were validated by using qRT-PCR. Monolayers transfected with the Vector-Ctrl were used for control. (C) Expression of type I IFN-controlled genes in IEC4.1 cells with deficient in *Ifnar1* in response to IFN-α stimulation. Cells were transfected with the Vector-NR_033736 for 24 h and then exposed to IFN-α stimulation for 2 h. Expression levels of type I IFN-controlled genes and selected type I IFN genes were validated. Data represent three independent experiments.

We then measured the effects of NR_033736 expression levels on type I IFN-controlled gene transcription in intestinal epithelium in response to IFN-α stimulation. Expression levels of type I IFN-controlled genes, including *Ifit1*, *Ifi44*, *Iigp1*, *Oas2*, and *Max2*, was globally further elevated in IFN-α-stimulated IEC4.1 cells treated with the NR_033736 siRNA, compared with that in IFN-α-stimulated IEC4.1 cells treated with the siRNA control (Fig 4A). Induction of type I IFN genes was observed in IEC4.1 cells following IFN-α stimulation; however, knockdown of NR_033736 with siRNA showed no obvious effects on the expression of type I IFN genes (Fig 4A). In contrast, expression levels of type I IFN-controlled genes, including *Ifit1*, *Ifi44*, *Iigp1*, *Oas2*, and *Max2*, were further elevated in IFN-α-stimulated IEC4.1 cells treated with the NR_033736 siRNA (Fig 4A). In addition, significant inhibition of IFN-α-induced expression of selected type I IFN-controlled genes was detected in IEC4.1 cells or murine 2D intestinal epithelial monolayers overexpressing NR_033736, compared with that in cells/monolayers transfected with the empty-vector control (Fig 4A and 4B). Expression levels of NR_033736 in IEC4.1 cells or murine 2D intestinal epithelial monolayers with or without IFN-α stimulation after transfection with the empty vector (Vector-Ctrl) or Vector-NR_033736 are shown in S5 Fig. Interestingly, the effects of NR_033736 overexpression on IFN-α-induced upregulation of type I IFN-controlled genes were not detected in IEC4.1 cells deficient in *Ifnar1* (Fig 4C). Of note, a higher level of Ifnb1 was detected in cells overexpressing NR_033736 either with or without IFN-α stimulation. Taken together, these data suggest that upregulation of NR_033736 can suppress type I IFN-controlled gene transcription in intestinal epithelial cells.

### Assembly of NR_033736 into the ISGF3 complex with the involvement of type I IFN signaling in intestinal epithelial cells

**Fig 5:**
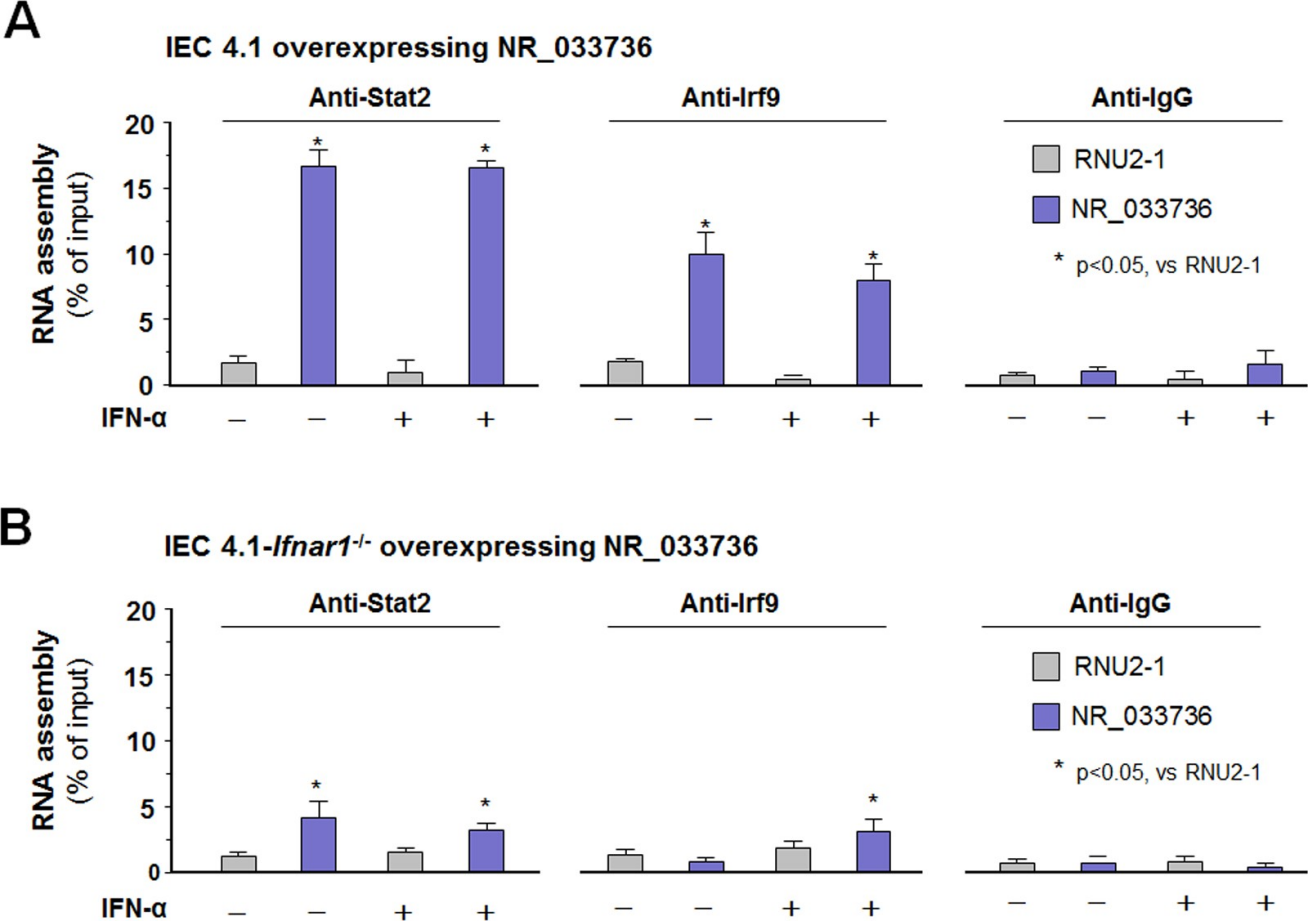
Assembly of NR_033736 into the ISG3 complex in intestinal epithelial cells. (A) Assembly of NR_033736 into the ISGF3 complex in IEC4.1 cells overexpressing NR_033736. Cells were transfected with the vector expression NR_033736 (Vector-NR_033736) for 24 h and cultured for additional 2 h in the presence or absence of IFN-α. RIP assay was carried out using antibodies to STAT2, IRF9, and IgG, respectively. NR_033736 was quantified by using qRT-PCR and an unrelated ncRNA (RNU2-1) was measured for control. (B) Assembly of NR_033736 into the ISGF3 complex in IEC4.1-*Ifnar1*^*-/-*^ cells overexpressing NR_033736. IEC4.1-*Ifnar1*^*-/-*^ cells were transfected with the Vector-NR_033736 or Vector-Ctrl for 24 h and cultured for additional 2 h in the presence or absence of IFN-α. RIP assay was carried out as described above. Data represent three independent experiments.

Type I IFNs signal to host cells by binding the conserved cell surface type I IFN receptor, IFN AR1/2. Ligation of IFNAR1/2 stimulates STAT2 tyrosine phosphorylation, leading to the recruitment of IRF9 and the formation of a STAT1/STAT2/IRF9 complex [known as the IFN-stimulated gene factor 3 (ISGF3) complex] to induce transcription of target genes [36, 37]. Interestingly, the expression levels of key components for the type I IFN signaling (e.g., STAT1/2/3, IRF9, IFNAR 1/2) were similar in IEC4.1 cells treated with the siRNA to NR_033736 compared with cells treated with the non-specific siRNA control (S6 Fig). Given the very low basal expression level of NR_033736 in IEC4.1 cells, we addressed the question whether NR_033736 can be assembled into the ISGF3 complex using IEC4.1 cells overexpressing NR_033736. Using the cross-linking RNA Immunoprecipitation (RIP) assay with antibodies to STAT2 or IRF9, we detected the assembly of NR_033736 into the ISGF3 complex in IEC4.1 cells overexpressing NR_033736 (Fig 5A). For control, no assembly of RNU2-1 (an unrelated ncRNA) was detected in the ISGF3 complex and no presence of NR_033736 was detected in the immunoprecipitates using a pan-mouse IgG specific antibody (Fig 5A). Of note, a similar amount of NR_033736 incorporated into the ISG3 complex was observed in cells in the presence and absence of IFN-α, probably due to the high level of NR_033736 in IEC4.1 cells overexpressing NR_033736 (S5 Fig). A much lower content of NR_033736 assembly to the ISGF3 complex was detected in IEC4.1 cells deficient in *Ifnar1* overexpressing NR_033736 (Fig 5B). These data indicate that NR_033736 can be assembled into the ISGF3 complex with the involvement of type I IFN signaling.

### NR_033736 may modulate type I IFN-mediated gene transcription through suppression of recruitment of the ISGF3 complex and attenuation of associated histone modifications at target gene loci

**Fig 6:**
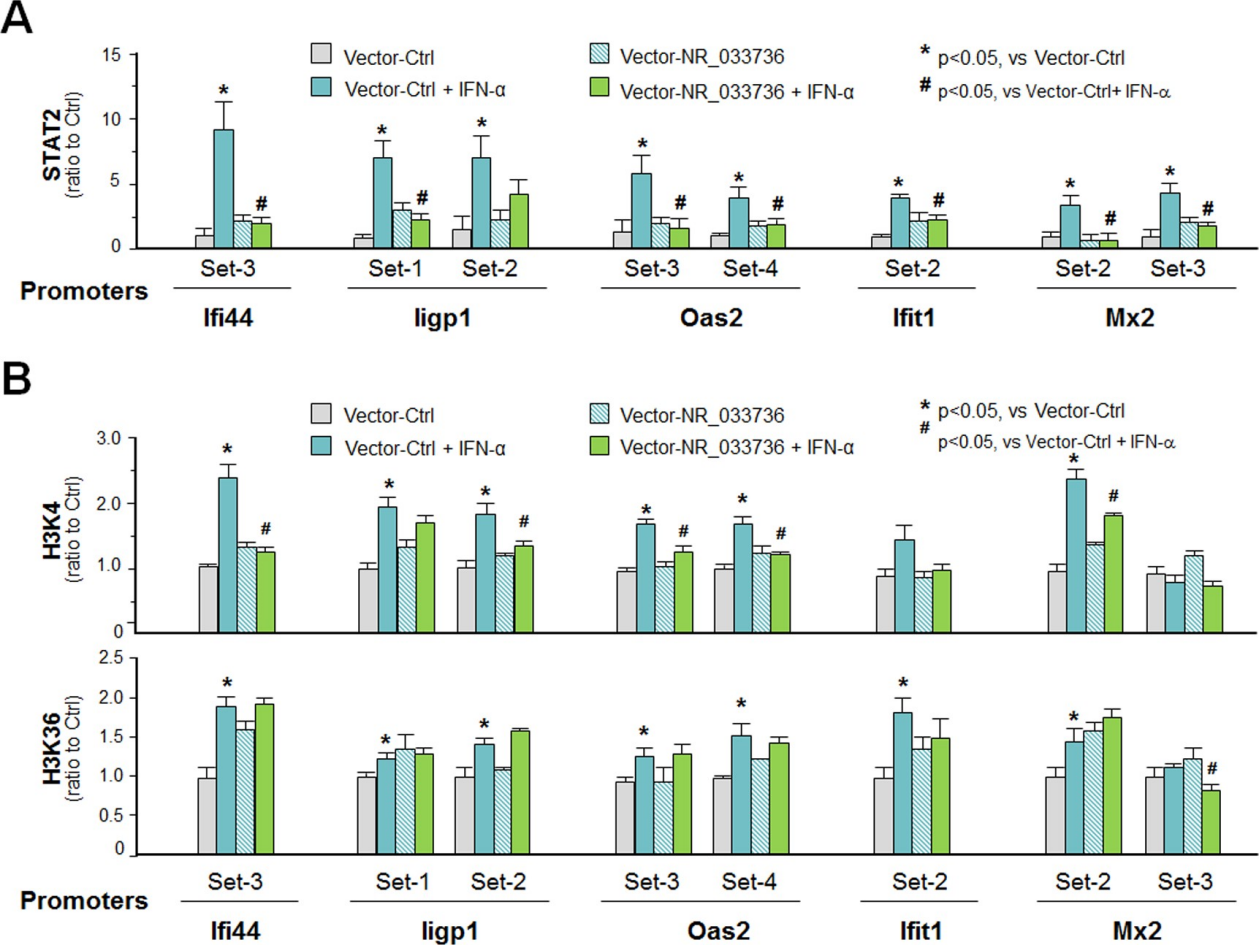
Impact of NR_033736 expression on the recruitment of STAT2 and enrichment of transcriptional active histone modifications at the promoter regions of type I IFN-controlled genes in IEC4.1 cells in response to Ifn-α stimulation. (A) Impact of NR_033736 expression on recruitment of STAT2 to the promoter regions of type I IFN-controlled genes in IEC4.1 cells in response to Ifn-α stimulation. Cells were transfected with the vector expression NR_033736 (Vector-NR_033736) or the empty vector (Vector-Ctrl) for 24 h and then exposed to Ifn-α stimulation for 2 h. Recruitment of STAT2 was measured using the ChIP assay with anti-STAT2 and PCR primers covering the known regions of the gene loci. (B) Impact of NR_033736 expression on enrichment of transcriptional active H3K4 and H3K36 methylations to the promoter regions of type I IFN-controlled genes in IEC4.1 cells in response to IFN-α stimulation. Cells were transfected with the Vector-NR_033736 or Vector-Ctrl for 24 h and then exposed to IFN-α stimulation for 2 h. Enrichment of H3K4 and H3K36 was measured using the ChIP assay with anti-H3K6 and H3K36, respectively, and PCR primers covering the known regions of the gene loci. Data represent three independent experiments.

To define how NR_033736 assembly may impact ISGF3-mediated type I IFN-controlled gene transcription, we performed chromatin immunoprecipitation (ChIP) analysis to measure the effects of NR_033736 assembly on the occupancy of ISGF3 complex to the gene loci of selected type I IFN-controlled genes. In consistent with results from previous studies [36, 37], a significant increase of ISGF3 complex (reflected by the recruitment of STAT2) was detected at the promoter regions of type I IFN-controlled gene loci (Figs 6A and S7). In contrast, overexpression of NR_033736 decreased ISGF3 occupancy to the promoter regions of these genes in IEC4.1 cells following IFN-α stimulation (Fig 6A). A significant increase of H3K4me3 and H3K36me3 occupancy to the corresponding promoter regions of these gene loci was detected in IEC4.1 cells following IFN-α stimulation (Fig 6B). Overexpression of NR_033736 decreased H3K4me3 occupancy, but not H3K36me3, to most of the corresponding promoter regions of these gene loci in IFN-α-treated IEC4.1 cells (Fig 6B). Therefore, NR_033736 may modulate type I IFN-mediated gene transcription through suppression of recruitment of the ISGF3 complex and inhibition of associated H3K4me3 modification at the gene loci.

### NR_033736 and a putative human ortholog can modulate intestinal epithelial innate defense against *C*. *parvum* infection

**Fig 7:**
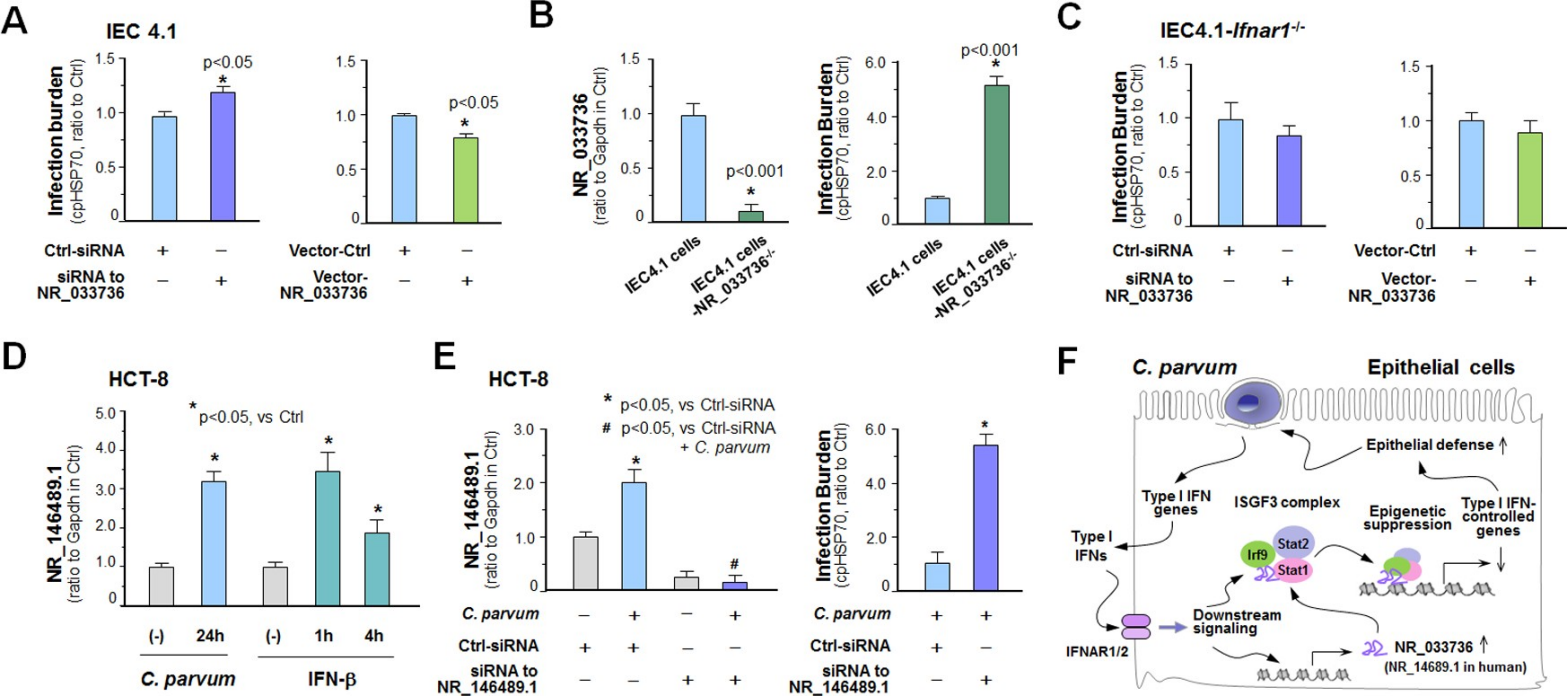
NR_033736 and its putative human ortholog modulate intestinal epithelial defense against *C*. *parvum* infection. (A) Effects of NR_033736 expression on the infection burden of *C*. *parvum* in IEC4.1 cells. Cells were treated with the siRNA to NR_033736 or transfected with the vector expressing NR_033736 (Vector-NR_033736) for 24 h and exposed to *C*. *parvum* infection for 24 h. Cells treated with the Ctr-siRNA were used as the control. Infection burden was quantified by measuring parasite cpHsp70 using qRT-PCR. (B) Knockout of NR_033736 via the CRISPR/Cas9 approach on *C*. *parvum* infection in IEC4.1 cells. IEC4.1 cells stably deficient in NR_033736 were generated via the CRISPR/Cas9 approach and verified by using qRT-PCR. Cells were exposed to *C*. *parvum* sporozoites for 24h and infection burden was quantified. (C) Effects of NR_033736 expression on the infection burden in IEC4.1 cells with deficient in *Ifnar1*. IEC4.1-*Ifnar1*^*-/-*^ *c*ells were treated with the siRNA to NR_033736 or transfected with the Vector-NR_033736 for 24 h and exposed to *C*. *parvum* infection for 24 h. Infection burden was quantified. (D) Upregulation of NR_146489.1 in cultured human intestinal epithelial cells (HCT-8) following *C*. *parvum* infection or IFN-β stimulation. HCT-8 cells were exposed to *C*. *parvum* infection for 24 h or treated with IFN-β for up to 4 h and expression level of NR_146489.1 was validated by using qRT-PCR. (E) Knockdown of NR_146489.1 increased the infection burden of *C*. *parvum* in HCT-8 cells. Cells were treated with the siRNA to NR_146489.1 for 24 h and exposed to *C*. *parvum* infection for additional 24 h. Cells treated with the non-specific scrambled siRNA were used as the control. Expression levels of NR_146489.1 and infection burden of *C*. *parvum* were quantified. (F) Model of NR_033736 in intestinal epithelial anti-*Cryptosporidium* defense. NR_033736 is upregulated in intestinal epithelial cells following *Cryptosporidium* infection through activation of the type I IFN signaling. NR_033736 can be assembled into the ISGF3 complex and suppresses type I IFN-controlled gene transcription. Therefore, upregulation of NR_033736 provides negative feedback regulation of type I IFN signaling and consequently, contributing to fine-tuning of epithelial innate defense against microbial infection. The putative human ortholog NR_146489.1 may share the same or similar functions.

Given the regulatory action of NR_033736 on type I IFN-mediated gene transcription, couple with the key role of type I IFN signaling in antimicrobial defense in general, we asked whether NR_033736 is involved in intestinal epithelial anti-*C*. *parvum* defense. To test this possibility, we genetically manipulated expression levels of NR_033736 in host cells and then measured its impact on host anti-*C*. *parvum* defense. Cells were collected at 4h after exposure to *C*. *parvum* for attachment/invasion assessment and at 24h post-infection for measurement of host defense (referred as infection burden), as previously reported [38]. Knockdown of NR_033736 with the designed siRNA, but not the non-specific siRNA, caused a significant increase in infection burden in IEC 4.1 cells (Fig 7A). We further took the CRISPR/Cas9 approach to establish stable IEC4.1 cells with deficient with NR_033736 (Fig 7B). Cells with deficient in NR_033736 showed a significant increase of infection burden (Fig 7B). Complementarily, overexpression of NR_033736 (926 nt sequence) in IEC4.1 cells resulted in a decrease of infection burden (Fig 7A). Moreover, siRNAs to knockdown NR_033736 also increased the burden of *C*. *parvum* infection in 2D intestinal monolayers derived from neonatal mice (S8 Fig). Overexpression of NR_033736 in 2D intestinal monolayers resulted in a decrease of infection burden (S8 Fig). Interestingly, knockdown or overexpression NR_033736 showed no changes in the attachment/invasion of IEC4.1 cells by *C*. *parvum* (S8 Fig). Moreover, knockdown or overexpression of NR_033736 showed no changes in *C*. *parvum* infection burden in IEC4.1 cells deficient in *Ifnar1* (Fig 7C).

While lncRNAs are generally with very low conservation of sequences between species as compared to protein coding genes (4,6), we identified several lncRNA gene candidates localized by the *DHX9 gene* in humans, including *NR_146489*.*1* and *NR_110793*.*1*, *NR_033302*.*2*, and *NR_148933*.*1* (S9 Fig). Upregulation of NR_146489.1, but not the others, was detected in cultured human intestinal epithelial HCT-8 cells following *C*. *parvum* infection (Figs 7D and S9). Upregulation of NR_146489.1 was also observed in HCT-8 cells following IFN-β stimulation (PeproTech, 50 IU/ml) (Fig 7D). Knockdown of NR_146489.1 with a designed siRNA, but not the non-specific siRNA, caused a significant increase in infection burden in HCT-8 cells (Fig 7E), suggesting that NR_146489.1 may be a putative human ortholog of NR_033736. Taken together, our data suggest that upregulation of NR_033736 is controlled by the type I IFN signaling and occurred in intestinal epithelial cells following *C*. *parvum* infection. NR_033736 can be assembled into the ISGF3 complex and suppresses type I IFN-mediated gene transcription. Therefore, upregulation of NR_033736 provides negative feedback regulation of type I IFN signaling through suppression of type I IFN-controlled gene transcription, and consequently, contributing to fine-tuning of epithelial innate defense against cryptosporidial infection (Fig 7F). The putative human ortholog NR_146489.1 may share the same or similar functions.

## Discussion

Recent advances have demonstrated a potential crucial role of lncRNAs in the regulation of inflammatory responses in various cell types and immunity in immune cells [7, 8]. The expression of many lncRNAs is tightly regulated by lineage-determining proteins and intracellular signaling pathways [6, 39]. In our previous study [23], we identified a panel of host cell lncRNAs that are upregulated in murine intestinal epithelial cells following *C*. *parvum* infection. NR_033736 is one of the top induced lncRNAs in *C*. *parvum*-infected host cells [23]. While some of these induced lncRNA genes are controlled by the TLR/NF-кB signaling pathway activated by *C*. *parvum* infection in infected host epithelial cells, such as lincRNA-Cox2 and NR_045064 [23], our data in this study support that upregulation of NR_033736 is mainly through activation of type I IFN signaling. Whereas expression of other lncRNA genes may be cell-type specific [6, 39], it appears that upregulation of NR_033736 occurs also in macrophages and microglia. Upregulation of a putative human ortholog is observed in human intestinal epithelial cells following *C*. *parvum* infection or IFN-α stimulation. Therefore, upregulation of NR_033736 may represent a general response in various cell types in response to type I IFN signaling activation.

Type I IFNs can be produced by almost every cell type, including intestinal epithelial cells [13, 36]. Type I IFNs signal to target cells by binding the conserved cell surface type I IFN receptor, IFNAR1/2. Ligation of IFNAR1/2 stimulates STAT2 tyrosine phosphorylation, leading to the recruitment of IRF9 and formation of the ISGF3 complex [13, 14, 36, 37]. The ISGF3 complex in turn migrates to the nucleus to bind to IFN-stimulated response elements (ISREs) and activate gene transcription [12–14]. Genes controlled through type I IFN signaling cover a broader physiological role in host defense and homeostasis, including regulation of innate and adaptive immune responses, responses to bacterial ligands, inflammasome activation, intestinal homeostasis and inflammatory and autoimmune diseases [13]. Whereas type I IFN signaling is protective in acute viral infections, it can have either protective or deleterious roles in bacterial infections and autoimmune diseases [40]. Therefore, to finely control the transcriptional activity by type I IFN signaling, cells have developed various feedback regulatory strategies to regulate type I IFN-stimulated gene transcription. Feedback regulatory mechanisms that suppress type I IFN-mediated responses include downregulation of cell surface IFNAR expression, induction of negative regulators, such as suppressor of cytokine signaling proteins and ubiquitin carboxy-terminal hydrolase 18 [41]. ISGF3-induced gene transcription can also be regulated by epigenetic factors and chromatin states at target gene loci or through the interaction of STATs with co-activators, corepressors, chromatin-modifying complexes and transcription elongation factors [40, 41].

Our findings indicate that induction of NR_033736 may provide additional negative feedback regulation to type I IFN-stimulated gene transcription. Specifically, NR_033736 may act as a co-regulator for the occupancy of ISGF3 complex to the promoter regions of type I IFN-targeted genes. Interestingly, NR_033736 can be assembled into the ISGF3 complex. Consequently, assembly of NR_033736 into the ISGF3 complex appears to suppress the recruitment of the complex to the promoter regions of target genes, resulting in suppression of active chromatin remodeling (H3K4me3) occupancy and consequently trans-suppression of type I IFN-controlled genes. Modulation of chromatin remodeling occupancy to regulate gene transcription by lncNRAs has been demonstrated in previous studies, including p300/MLL-associated chromatin remodeling by NR_045064 [23], Hmgb1-associated histone modifications by lincRNA-Tnfaip3 [11], and SWI/SNF complex-mediated gene transcription by lincRNA-Cox2 [9]. It is unclear how NR_033736 is assembled to the ISGF3 complex and how type I IFN signaling may regulate NR_033736 assembly. Of note, cells overexpressing NR_033736 were used in our assembly analysis due to the very low basal expression level of NR_033736 in the cells. Identification of the RNA-binding partner(s) in the ISGF3 complex for NR_033736 assembly and clarification of its association with type I IFN signaling in future studies should provide answers to this question.

Given the importance of type I IFN signaling in regulating intestinal epithelial homeostasis and immunity [12, 13], it is plausible to speculate that feedback regulation of type I IFN signaling by NR_033736 may be involved in the regulation of intestinal epithelial antimicrobial defense. Particularly, activation of type I IFN signaling in infected epithelial cells may represent an important element of intestinal epithelial anti-*C*. *parvum* defense [20, 21]. It was reported previously that pre-treatment of cultured epithelial cells with IFN-α before exposure to *C*. *parvum* can modestly inhibit parasite invasion of host cells *in vitro* [42]. However, we observed a higher infection burden (reflecting a weaker epithelial defense) in cells with the knockdown of NR_033736 through siRNA. Complementarily, a lower infection burden was detected in cells overexpressing NR_033736. Knockdown or overexpression NR_033736 showed no impact on the attachment/invasion of host cells by *C*. *parvum*. Therefore, upregulation of NR_033736 promotes intestinal epithelial anti-*C*. *parvum* defense.

We speculate that NR_033736 may modulate intestinal anti-*C*. *parvum* defense through type I IFN-dependent and/or type I IFN-independent mechanisms. *Cryptosporidium* infection triggers a strong type I IFN response in infected intestinal epithelium [22]. Release of type I IFNs can stimulate epithelial cell function through ligation of receptors expressed on themselves in an autocrine manner. Given the higher infection burden and a stronger type I signaling in cells with the NR_033736 knockdown, it is possible that a strong type I IFN response in infected intestinal epithelial cells may actually impair epithelial anti-*C*. *parvum* defense. Future investigation should address this possibility and indeed, the production of type I IFNs could serve as a double-edged sword: type I IFNs provide early resistance against acute viral infections but are detrimental to the host during certain bacterial, parasitic, and chronic viral infections [43–45].

Besides type I IFN-controlled genes, we cannot exclude the possibility that NR_033736 may influence the expression of other defense genes and thus, modulate host anti-cryptosporidial immunity through type I IFN-independent mechanisms. Modulation of type I IFN signaling by NR_033736 may subsequently affect intestinal epithelial cell response to IFN-γ or other effect molecules from immune cells at the intestinal epithelium to modulate anti-*C*. *parvum* defense. Cross-link network has recently been demonstrated in various cell types between type I IFN signaling and other intracellular pathways such as IFN-γ signaling and MAPK signaling pathways [13, 20, 37]. IFN-γ is key to mucosal innate anti-*C*. *parvum* defense [27, 46, 47]. Nevertheless, IFN-γ is mainly produced by activated T cells and NK cells and other innate lymphoid cells at intestinal mucosa [48, 49]. We detected the impact of genetic manipulation of NR_033736 expression on intestinal epithelial anti-*C*. *parvum* defense using *in vitro* infection cultures and *ex vivo* 2D monolayer infection models. IFN-γ was absent in our *in vitro* infection cultures and these cell types producing IFN-γ were not present in the 2D monolayer and *in vitro* models.

In summary, our data suggest that NR_033736 provides negative feedback regulation of type I IFN signaling through suppression of type I IFN-controlled gene transcription, and consequently, contributing to fine-tuning of epithelial cell innate defense against cryptosporidial infection. Given the fact that upregulation of NR_033736 (and the putative human ortholog NR_146489.1) is a global cellular response to IFN-α stimulation, further mechanistic investigation should address their regulatory role in the fine-tuning of type I IFN signaling in other cell types and in host immunity to microbial infection in general.

## Materials and methods

### Ethics statement

This study was carried out in strict accordance with the recommendations in the Guide for the Care and Use of Laboratory Animals of the National Institutes of Health under the Assurance of Compliance Number A3348-01. All animal experiments were done in accordance with procedures (protocol number #0959) approved by the Institutional Animal Care and Use Committee of Creighton University.

#### *C*. *parvum* and cell lines

*C*. *parvum* oocysts of the Iowa strain were purchased from a commercial source (Bunch Grass Farm, Deary, ID). The intestinal epithelial cell line (IEC4.1) was received as a kind gift from Dr. Pingchang Yang (McMaster University, Hamilton, Canada). The murine intestinal epithelial cell line, mulNTEPI cells, was purchased from InSCREENeX Cellular Screening Technologies (Germany). The BV2 mouse microglia cells and RAW264.7 mouse macrophage cells were obtained from ATCC (Manassas, VA, USA). HCT-8 cells were purchased from ATCC (Manassas, VA). Culture media were supplied with 10% FBS (Ambion) and antibiotics (100 IU/ml of penicillin and 100 μg/ml of streptomycin). Stable IEC4.1 cells with deficient in *Ifnar1* or *NR_033736* were generated through transfection of cells with the CRISPR/Cas9 KO^(h)^ (NR_033736-CRISPR/Cas9 KO and Ifnar1-CRISPR/Cas9 KO, respectively) and the HDR plasmid (NR_033736-HDR and Ifnar1-HDR plasmid, respectively), as previously described [33].

#### Infection models and infection assays

Models of intestinal cryptosporidiosis using intestinal epithelial cell lines and enteroids were employed as previously described [23, 29]. Intestinal epithelium and 3D enteroids were isolated and cultured as previously described [29]. Briefly, small intestines were opened longitudinally and washed with ice-cold Ca^2+^ and Mg^2+^ free PBS, then were cut into 1–2 mm fragments and washed with ice-cold Ca^2+^ and Mg^2+^ free PBS 3 times. The cut fragments were incubated in ice-cold 2 mM PBS/EDTA at 4°C for 30 min with gentle rotation followed by vigorous shake until the PBS solution was mostly opaque with dislodged crypt and villus particles. Large tissue fragments were removed through a 100-μm cell strainer (Becton-Dickinson Bioscience, Franklin Lakes, NJ). The pass through was centrifuged 150g for 5 min at 4°C and the pellet was collected as the intestinal epithelium. 2D monolayers derived from 3D enteroids were cultured as previously described [29–31] and exposed to *C*. *parvum* infection for 24–48 h.

A well-developed infection model of cryptosporidiosis in neonatal mice was used for *in vivo* experiments [27, 28]. Mice at the age of 5 days after birth received *C*. *parvum* oocysts by oral gavage (10^5^ oocysts per mice). Mice receiving vehicle (PBS) by oral gavage were used as control. At 24 and 48h after *cryptosporidium* or vehicle administration, animals were sacrificed, and ileum intestine tissues were collected. At least five animals from each group were sacrificed and ileum epithelium tissues were obtained for biochemical analyses and infection assessment, as previously reported [23, 29, 33, 34].

#### qRT-PCR, RNA-Seq, and western blot

For quantitative analysis of RNA expression, qRT-PCR was performed as previous reported [9–11, 33, 34, 50] using the SYBR Green PCR Master Mix (Applied Biosystems, Carlsbad, CA). The sequences for all the primers described above are listed in S3 Table. For RNA-seq, total RNAs were isolated with the RNA isolation Kit (Upstate Biotechnologies) and sequence (BGISeq500) and data analysis were performed by the BGI Americas Corporation (Cambridge, MA). Western blot was performed as previously reported [33]. The following antibodies were used for blotting: anti-Igtp (Santa Cruz Biotechology), anti-Iigp1(MyBioSource), and anti-Usp18 (Gentaur), and anti-Gapdh (Abcam).

#### siRNAs and plasmids

Custom-designed RNA oligos against NR_033736 and a scrambled RNA were synthesized by Exiqon and transfected into cells with Lipofectamine RNAimax according to the manufacturer’s protocol (Invitrogen). Sequences of siRNAs are: GUGCUUGUUAAGCGAAACUUU (sense) and UUCACGAACAAUUCGCUUUGA (antisense) for NR_033736, and GAGCCAAGAUGGCCAAAUAUU (sense) and UUCUCGGUUCUACCGGUUUAU (antisence) for NR_146489.1. A non-specific scrambled sequence UUCUCCGAACGUGUCACGUUU (sense) and ACGUGACACGUUCGGAGAAUU (antisense) for the control. For NR_033736 siRNA transfection into enteroids, the electroporation approach was used with the Neon transfection system (Thermo Fisher Scientific, Waltham, MA; electroporation parameter: 1600 v, 10 ms, pulse 3). To generate the NR_033736 expressing vector, the 926 nt sequence of NR_033736 was cloned into the pTarget vector according to the manufacturer’s protocol. LncRNA-NR_033736-pTarget was transfected to cells with Lipofectamine 2000 (Invitrogen). The Ifnar1-CRISPR/Cas9 KO and Ifnar1-HDR plasmids were obtained from Santa Cruz. The primers and the guide RNA sequence used to generate the NR_033736-CRISPR/Cas9 KO and NR_033736-HDR plasmids are listed in S3 Table.

#### Luciferase reporter constructs and luciferase assay

The -2000 to +0 sequence (genome site: chr1:155261364–155263363 in the NCB137/mm9 assembly) of the *NR_033736* (*E330020D12Rik)* gene locus was cloned into the multiple cloning site of the pGL3-Basic Luciferase vector (Promega Corporation). The following primers were used to amplify the sequence: 5’- GGCTAGCAAAGGACACACAGACTTAC -3’ (forward, with the restriction site for *NheI*) and 5’- CAAGCTTCTTATATACACCCCAGCAC -3’ (reverse, with the restriction site for *HindIII*). Cultured cells were transfected with the reporter construct overnight and then exposed to *C*. *parvum* infection for 16h or IFN-α stimulation for 8h. The pGL3 basic vector (the empty vector without the *NR_033736* promoter) was used as control. The luciferase activity was normalized to the control β-galactosidase level and compared with that of the empty vector.

#### RNA stability

RNA stability assay was performed by real-time PCR as previously reported [51]. Briefly, cells were transfected with the NR_033736 expressing vector or the empty control vector for 24h and transcription was then blocked using actinomycin D (10 μg/ml, Sigma); RNAs were isolated at various time points after actinomycin D treatment. Real-time PCR was then performed using 500 ng of template cDNA for each mRNA gene of interest. Experiments were performed in triplicate. The relative abundance of each mRNA was calculated using the ΔΔCt method and normalized to Gapdh. The relative amount of mRNA at 0h following actinomycin D treatment was arbitrarily set to 1. Curve fittings of the resultant data were performed using Microsoft Excel and the half-lives of the RNAs calculated.

#### RIP and ChIP analyses

The formaldehyde crosslinking RIP was performed as described [52]. Briefly, cells in culture were first treated with trypsin, washed once with culture medium containing 10% FBS, washed twice with 10 ml PBS, and resuspended in 10 ml of PBS. Formaldehyde (37% stock solution) was then added to a final concentration of 0.3% (v/v) and incubated at room temperature for 10 min with slow mixing. Crosslinking reactions were quenched by the addition of glycine (pH7.0) to a final concentration of 0.25 M followed by incubation at room temperature for 5 min. The cells then harvested by centrifugation using a clinical centrifuge at 3000 rpm (237g) for 4 min followed by two washes with ice-cold PBS. Cell pellets were resuspended in 1 ml of lysis buffer (10 mM Tris–HCl pH 7.4, 10 mM NaCl, 3 mM MgCl_2_, 0.5% NP-40 and cocktail protease inhibitor and RNase inhibitor 100 unit). After a 10-min incubation on ice, nuclei were collected by centrifugation (500 g, 5 min) and washed with lysis buffer devoid of NP-40. After centrifugation, the pellets were resuspended in 100 μl nuclei lysis buffer (10 mM Tris–HCl pH 7.4, 400 mM NaCl, 1 mM EDTA, 1 mM DTT and cocktail protease inhibitor plus RNase inhibitor 10 unit) and mixed thoroughly for 30 min at 4°C. The nuclei lysates were diluted 5-fold in WCE buffer (20 mM HEPES, pH 7.4, 0.2 M NaCl, 0.5% Triton X-100, 10% glycerol, 1 mM EDTA, 1 mM EGTA, 10 mM β-glycerophosphate, 2 mM Na3VO4, 1 mM NaF, 1 mM DTT, cocktail protease inhibitor plus RNase inhibitor). Solubilization of crosslinked complexes was done by mechanical sonication by three rounds of sonication, 20s each, in a Microson XL2007 ultrasonic homogenizer with a microprobe at an amplitude setting of 5 (output, 10–12 W). Insoluble materials were removed by microcentrifugation at 14,000 rpm (16,000g) for 20 min at 4°C. Preclearing lysates with 20 μl of PBS washed Magna CHIP Protein A+G Magnetic Beads (Millipore, Massachusetts). The precleared lysate (250 μl) was then diluted with WCE buffer (250 μl), mixed with the specific antibody-coated beads, and incubated with rotation at 4°C for 4h, followed by 4 times washing with WCE buffer containing protease and RNase inhibitors. The collected immunoprecipitated RNP complexes and input were digested in RNA PK Buffer pH 7.0 (100 mM NaCl, 10 mM TrisCl pH 7.0,1 mM EDTA, 0.5% SDS) with addition of 10 μg Proteainse K and incubated at 50°C for 45 min with end-to-end shaking at 400 rpm. Formaldehyde cross-links were reversed by incubation at 65°C with rotation for 4h. RNA was extracted from these samples using Trizol according to the manufacturer’s protocol (Invitrogen Corp.) and treated with DNA-free DNase Treatment & Removal I kit according to the manufacturer’s protocol (Ambion Inc., Austin, TX). The presence of RNA was measured by quantitative, strand-specific RT-PCR using the iCycler iQ Real-time detection system (BioRad). Gene-specific PCR primer pairs are listed in S3 Table. Anti-STAT2 (Cell Signaling Technology) and anti-IRF9 (Proteintech) were used for ChIP analysis. PCR primer sequences for RIP and ChIP analysis are listed in the S3 Table.

#### Statistical analysis

All values are given as mean + S.E. Means of groups were from at least three independent experiments and compared with Student’s *t* test (unpaired) or the ANOVA test when appropriate. *p* values < 0.05 were considered statistically significant.

## Supporting information

S1 Fig Expression levels of selected type I IFN-controlled genes in RAW264.7 and BV2 cells following IFN-α stimulation. RAW264.7 (**A**) and BV2 (**B**) cells were exposed to IFN-a stimulation for up to 24 h and expression levels of Ifi44, Iift1, and Mx2 genes were validated by using real-time quantitative PCR. Data represent three independent experiments. *P < .05 and ** P < .01 vs cells of the non-IFN-α stimulation control.
(TIF)

S2 Fig Generation of stable IEC4.1 cells with deficient in Ifnar1 using the CRISPR/Cas9 approach. Stable IEC4.1 cells with deficient in *Ifnar1* were generated through transfection of cells with the Ifnar1-CRISPR/Cas9 KO^(h)^ and the Ifnar1-HDR plasmids. Deletion was verified by using PCR primers covering the designed regions of Ifnar1.
(TIF)

S3 Fig Generation of NR_033736 expressing vector to overexpress NR_033736 in IEC4.1 cells. (**A**) the 926 nt sequence of NR_033736 is shown. The sequence was cloned into the pTarget vector. (**B**) Overexpression of NR_033736 in IEC4.1 cells. Cells were transfected with the vector expressing NR_033736 (Vector-NR_033736) for 24h and level of NR_033736 were measured. Cells transfected with the empty vector (Vector-Ctrl) were used as the control. Data represent three independent experiments. *P < .05 vs cells of the non-infected control.
(TIF)

S4 Fig Impact of NR_033736 expression on Ifit1 RNA stability. IEC4.1 cells were transfected with the NR_033736 expressing vector (Vector-NR_033736) for 24h and then treated with actinomycin D to block transcription. At 0, 2, 4, 6h after actinomycin D treatment, cells were collected and levels of Ifit1 RNA expression were measured. Cells transfected with the empty vector (Vector-Ctrl) were used as the control. Data represent three independent experiments.
(TIF)

S5 Fig Expression levels of NR_033736 in IEC4.1 cells or murine 2D intestinal epithelial monolayers with or without IFN-α stimulation after transfection with the empty vector (Vector-Ctrl) or the vector expressing NR_033736 (Vector-NR_033736). The crypt units of small intestinal epithelium from neonates of 5 days old were isolated and cultured into 2D monolayers. IEC4.1 cells and murine 2D intestinal epithelial monolayers were transfected with either the Vector-Ctrl or Vector-NR_033736 for 24 h and then exposed to IFN-α stimulation for 2 h. Expression levels of NR_033736 in IEC4.1 cells (**A**) and 2D monolayers (**B**) were validated by using qRT-PCR. Data represent three independent experiments.
(TIF)

S6 Fig Heat map representing expression levels of the genes coding the key elements for the type I IFN signaling in IEC4.1 cells following *C*. *parvum* infection. IEC4.1 cells were exposed to *C*. *parvum* infection for 24 h followed by genome-wide array analysis. Heat map shows the expression levels of these genes coding the key elements for the type I IFN signaling in infected IEC4.1 cells vs non-infected control cells.
(TIF)

S7 Fig Recruitment of STAT2 to the promoter regions of type I IFN-controlled genes in IEC 4.1 cells following IFN-α stimulation. Cells were treated with IFN-α for 1 h and then processed for ChIP analysis using anti-STAT2 and PCR primers targeting the designed promoter regions of each gene. Data represent three independent experiments. *P < .05 and ** P < .01 vs cells of the non-IFN-α stimulation control.
(TIF)

S8 Fig NR_033736 modulates intestinal epithelial defense against *C*. *parvum* infection. (**A**) Knockdown of NR_033736 increased the infection burden of *C*. *parvum* in IEC4.1 cells. Cells were treated with the siRNA to NR_033736 (siRNA-NR_033736) for 24 h and exposed to *C*. *parvum* infection for 8 and 24 h. Cells treated with the non-specific scrambled siRNA (Ctrl-siRNA) were used as the control. Infection burden of *C*. *parvum* was quantified by measuring parasite cpHsp70 or cp18s using qRT-PCR. (**B**) overexpression of NR_033736 decreased the infection burden of *C*. *parvum* in IEC4.1 cells. Cells were transfected with the empty vector (Vector-Ctrl) or vector expressing NR_033736 (Vector-NR_033736) for 24 h and exposed to *C*. *parvum* infection for 8 and 24 h. Infection burden of *C*. *parvum* was quantified. (**C**) Effects of NR_033736 expression on the infection burden of *C*. *parvum* in murine 2D intestinal epithelial monolayers. The crypt units of small intestinal epithelium from neonates of 5 days old were isolated and cultured into 2D monolayers. Epithelial monolayers were then treated with the siRNA-NR_033736 or transfected with the Vector-NR_033736 for 24 h and exposed to *C*. *parvum* infection for 24 h. Infection burden of *C*. *parvum* was quantified. (**D**) NR_033736 on the attachment and invasion of *C*. *parvum* to IEC4.1 cells. Cells were treated with the siRNA-NR_033736 or transfected with the Vector-NR_033736 for 24 h and then exposed to *C*. *parvum* sporozoites for 4h (time frame for attachment and invasion). Cells treated with the Ctrl-siRNA or transfected with the Vector-Ctrl were used as the control and *C*. *parvum* infection was quantified. Data represent three independent experiments. *P < .05 vs cells treated with Ctrl-siRNA or Vector-Ctrl.
(TIF)

S9 Fig Upregulation of NR_146489.1, a putative human orthologs of NR_033736, in HCT-8 cells following *C*. *parvum* infection. Four lncRNA gene candidates were identified by the *DHX9* gene in the human genomic database. Upregulation of NR_146489.1, but not the other three candidates, was detected in HCT cells after exposure to *C*. *parvum* infection for 24h (**A**). Data represent three independent experiments. The chromatin localization of these lncRNA candidates and the sequence of NR_146489.1 are listed in (**B**).
(TIF)

S1 Table List of type I IFNs and type I IFN-controlled genes in IEC4.1 cells following *C*. *parvum* infection (Excel file). (XLSX)

S2 Table List of genes whose expression levels are altered in NR_033736-siRNA treated IEC4.1 cells following *C*. *parvum* infection (Excel file). (XLSX)

S3 Table List of primers used for PCR, ChIP and for generating constructs, and probe sequences for ChIRP analysis (Excel file). (XLSX)
