# Supplementary figures and images for: A host cell long noncoding RNA NR_033736 regulates type I interferon-mediated gene transcription and modulates intestinal epithelial anti-Cryptosporidium defense
Source: PLoS Pathog. 2021 Jan 22;17(1):e1009241. doi: 10.1371/journal.ppat.1009241 (PMC7857606; doi:10.1371/journal.ppat.1009241)

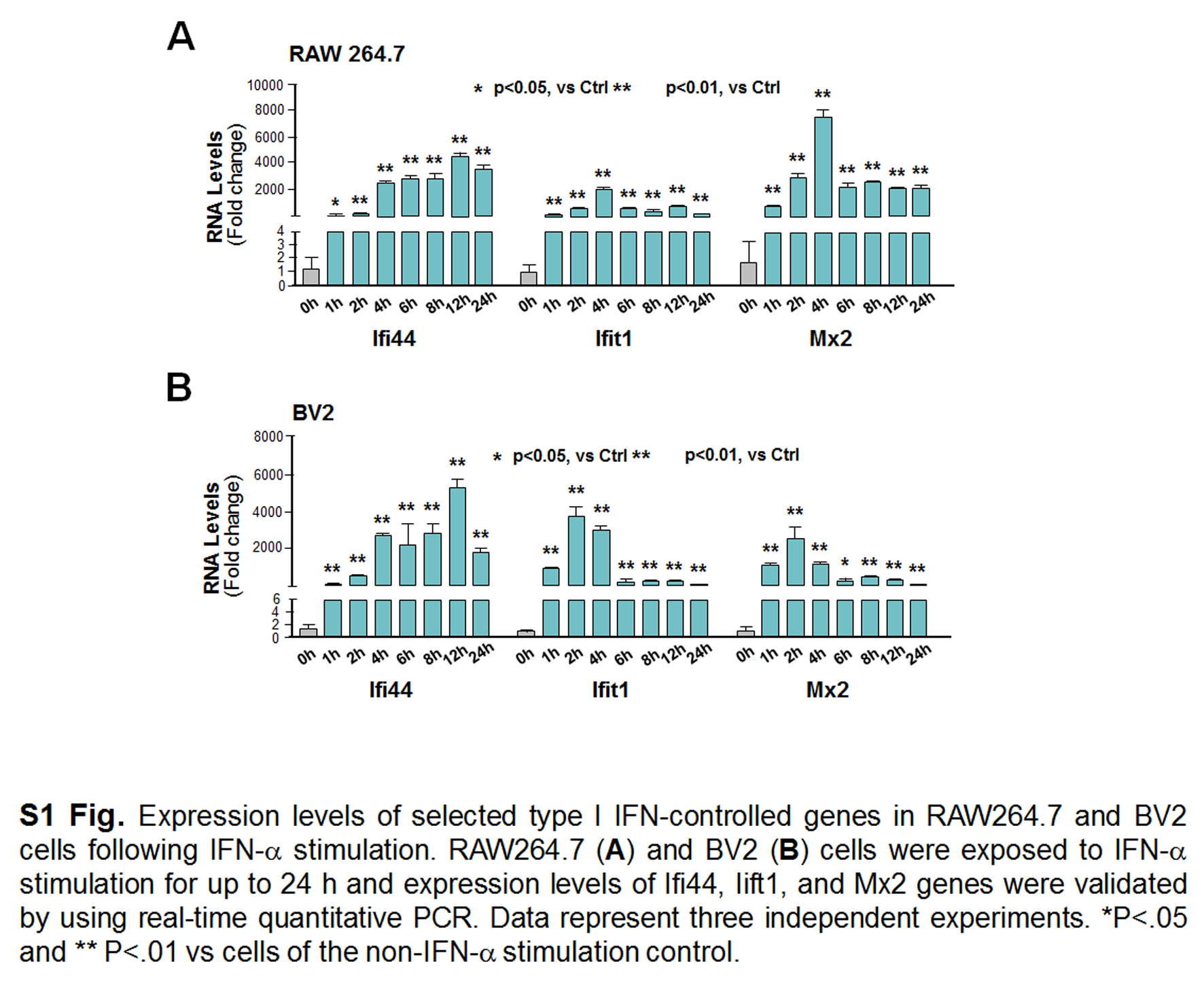

Supplement: S1 Fig — RAW264.7 (A) and BV2 (B) cells were exposed to IFN-a stimulation for up to 24 h and expression levels of Ifi44, Iift1, and Mx2 genes were validated by using real-time quantitative PCR. Data represent three independent experiments. *P < .05 and ** P < .01 vs cells of the non-IFN-α stimulation control. (TIF) [file ppat.1009241.s001.tif]

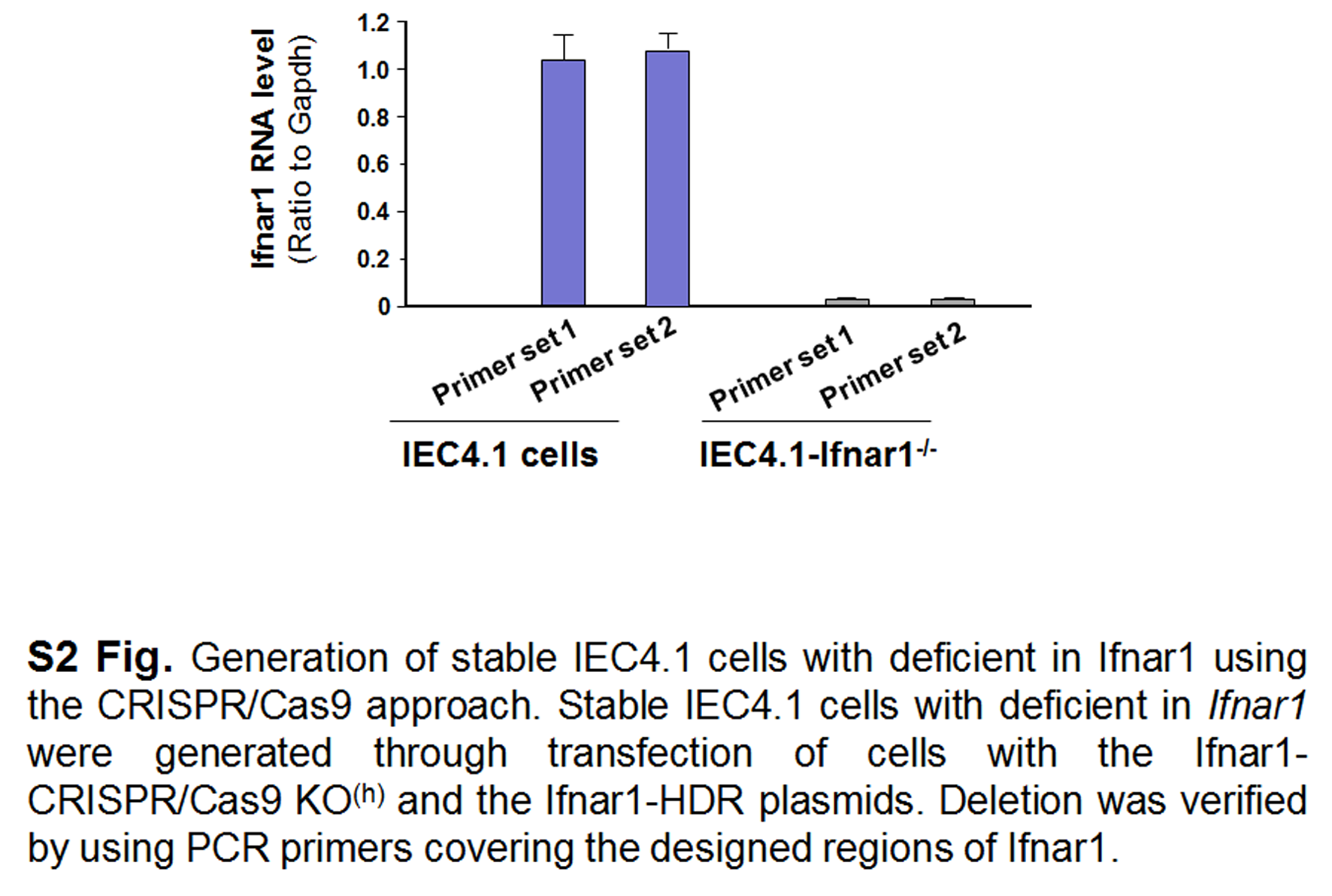

Supplement: S2 Fig — Stable IEC4.1 cells with deficient in Ifnar1 were generated through transfection of cells with the Ifnar1-CRISPR/Cas9 KO(h) and the Ifnar1-HDR plasmids. Deletion was verified by using PCR primers covering the designed regions of Ifnar1. (TIF) [file ppat.1009241.s002.tif]

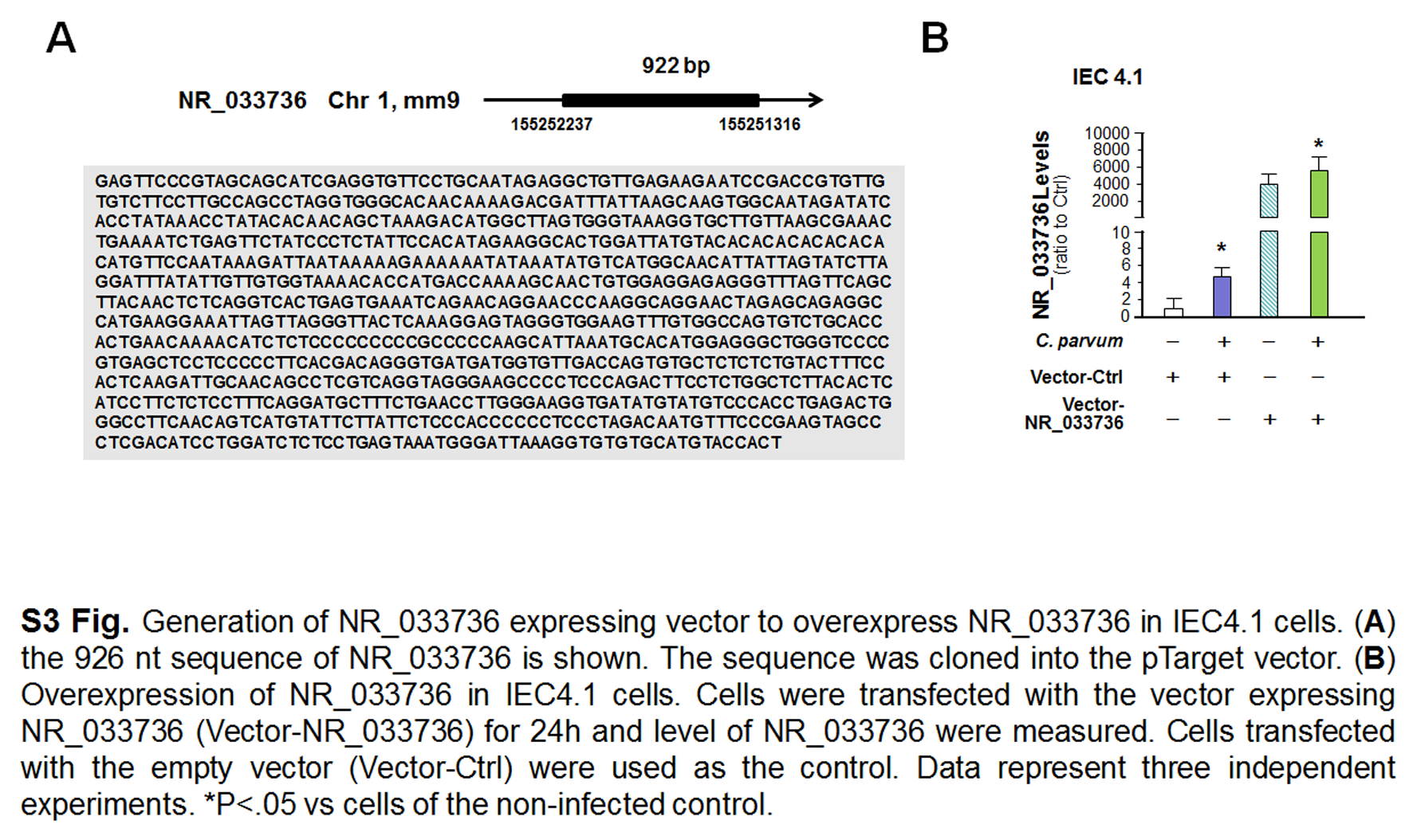

Supplement: S3 Fig — (A) the 926 nt sequence of NR_033736 is shown. The sequence was cloned into the pTarget vector. (B) Overexpression of NR_033736 in IEC4.1 cells. Cells were transfected with the vector expressing NR_033736 (Vector-NR_033736) for 24h and level of NR_033736 were measured. Cells transfected with the empty vector (Vector-Ctrl) were used as the control. Data represent three independent experiments. *P < .05 vs cells of the non-infected control. (TIF) [file ppat.1009241.s003.tif]

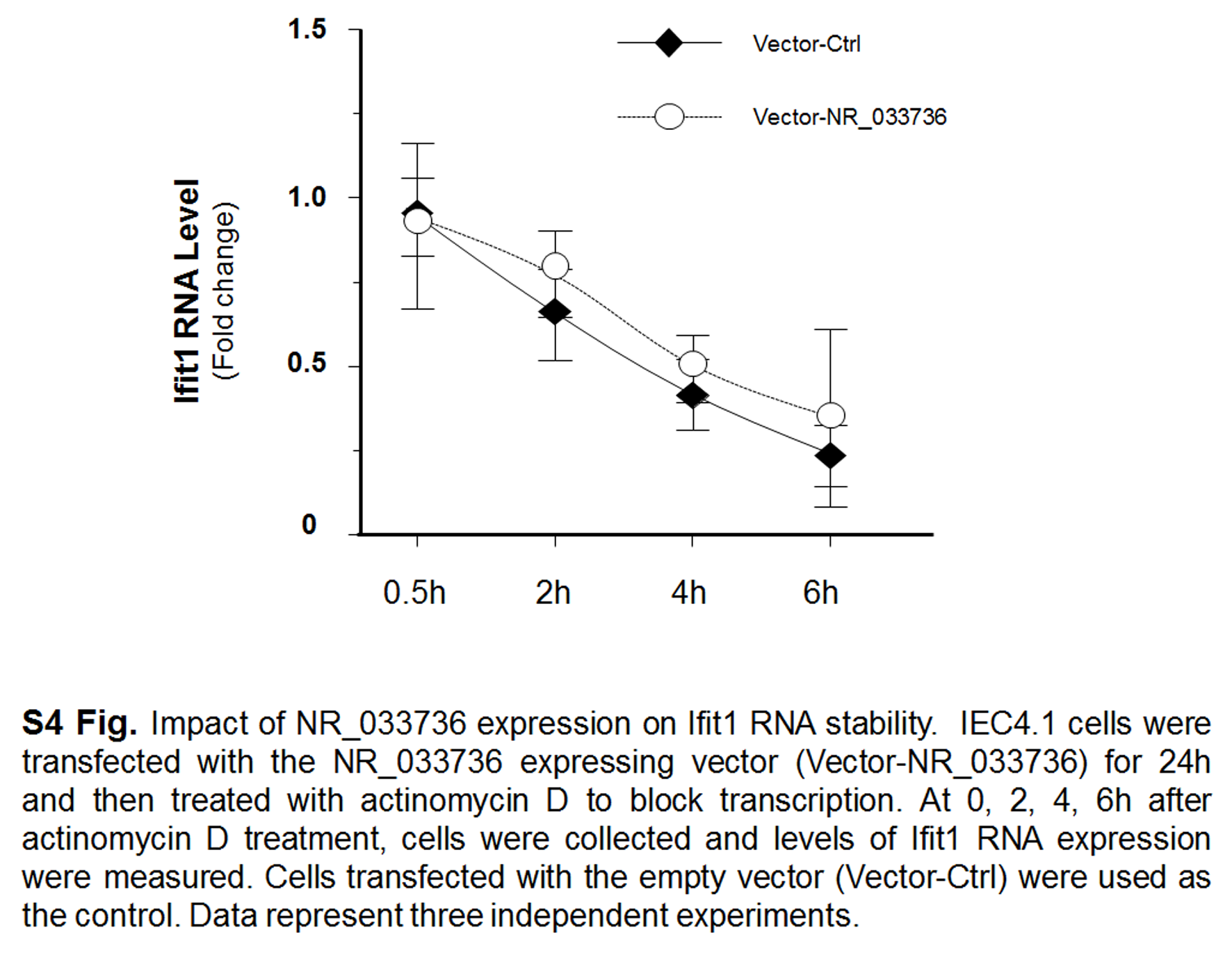

Supplement: S4 Fig — IEC4.1 cells were transfected with the NR_033736 expressing vector (Vector-NR_033736) for 24h and then treated with actinomycin D to block transcription. At 0, 2, 4, 6h after actinomycin D treatment, cells were collected and levels of Ifit1 RNA expression were measured. Cells transfected with the empty vector (Vector-Ctrl) were used as the control. Data represent three independent experiments. (TIF) [file ppat.1009241.s004.tif]

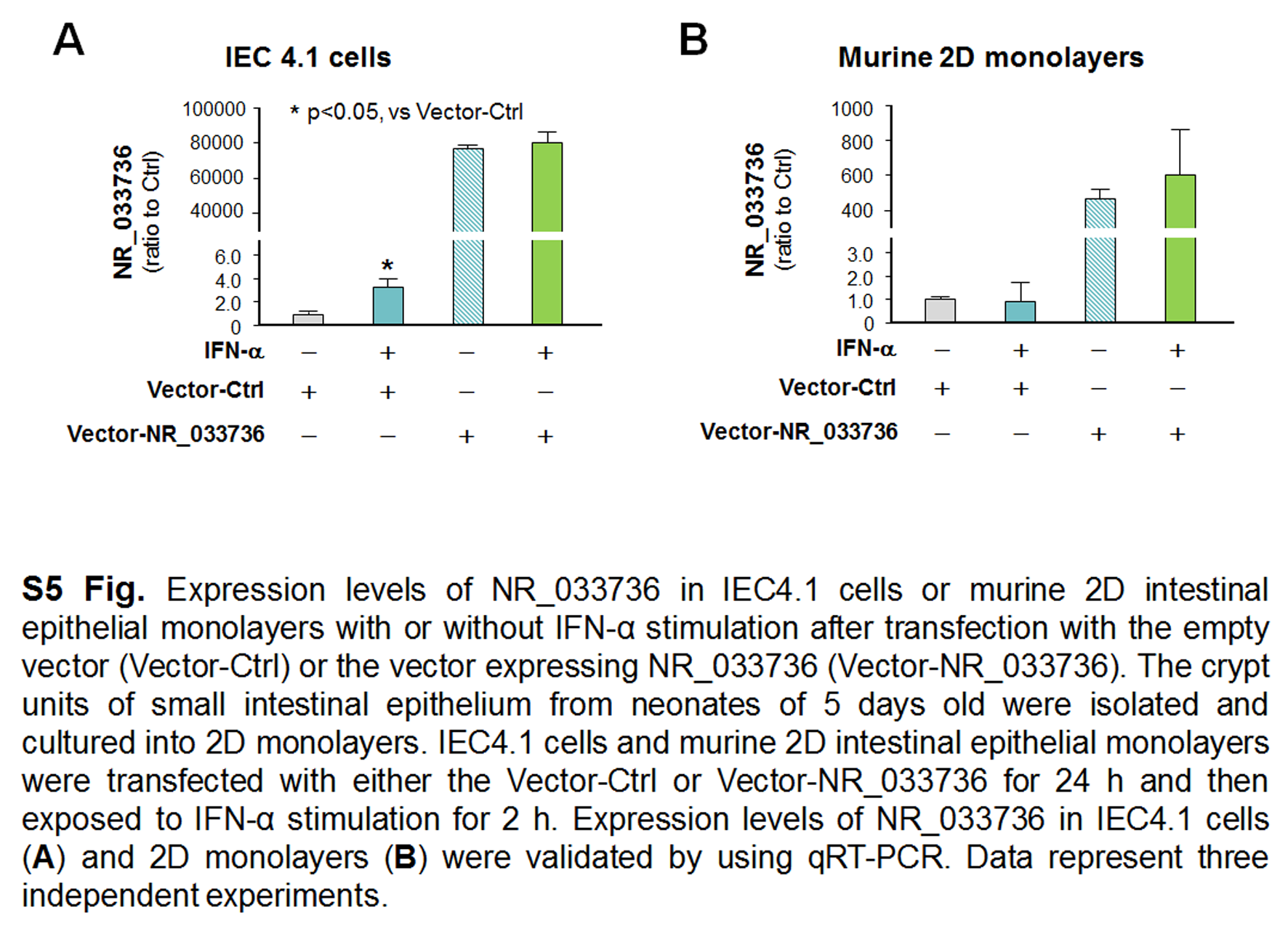

Supplement: S5 Fig — The crypt units of small intestinal epithelium from neonates of 5 days old were isolated and cultured into 2D monolayers. IEC4.1 cells and murine 2D intestinal epithelial monolayers were transfected with either the Vector-Ctrl or Vector-NR_033736 for 24 h and then exposed to IFN-α stimulation for 2 h. Expression levels of NR_033736 in IEC4.1 cells (A) and 2D monolayers (B) were validated by using qRT-PCR. Data represent three independent experiments. (TIF) [file ppat.1009241.s005.tif]

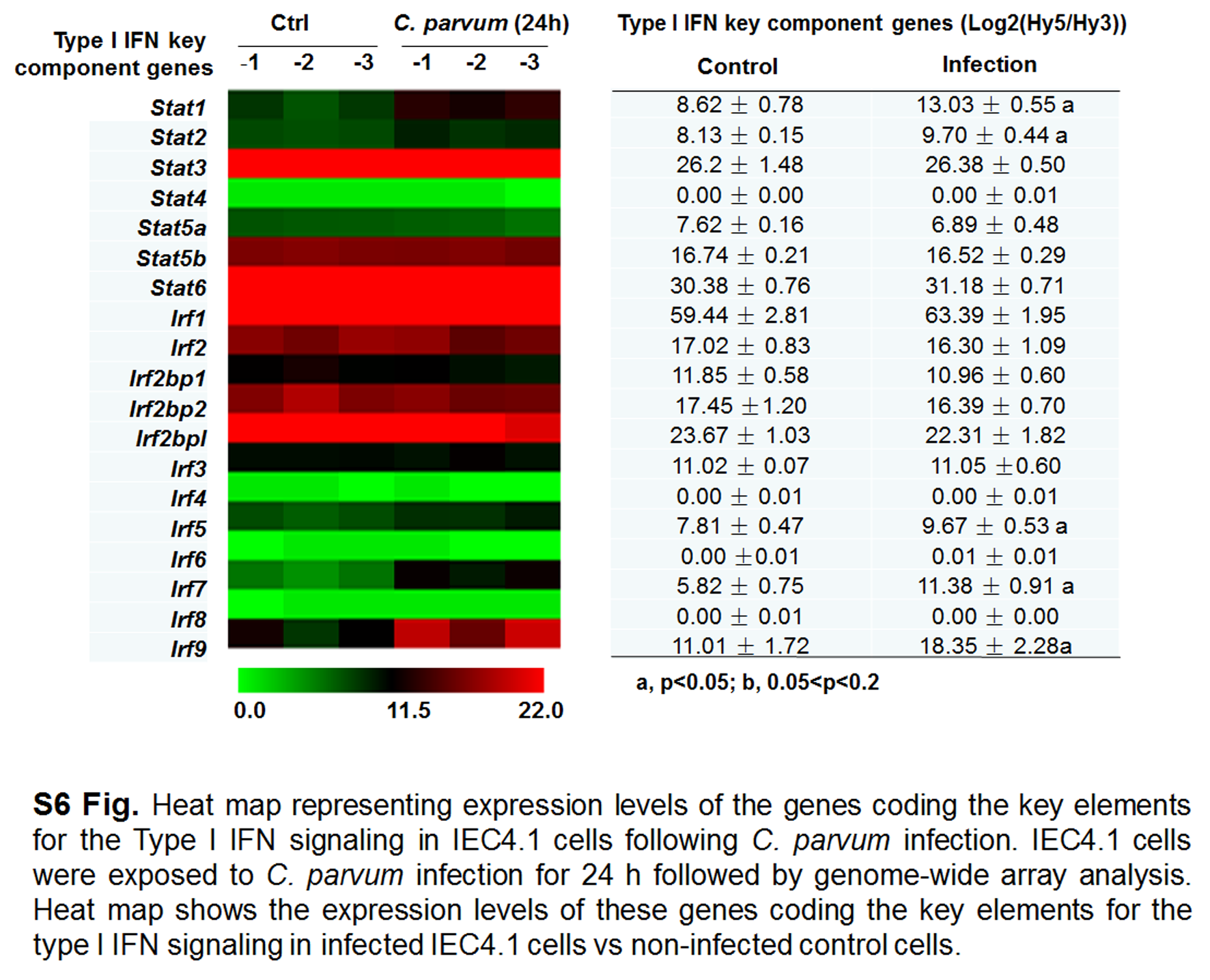

Supplement: S6 Fig — IEC4.1 cells were exposed to C. parvum infection for 24 h followed by genome-wide array analysis. Heat map shows the expression levels of these genes coding the key elements for the type I IFN signaling in infected IEC4.1 cells vs non-infected control cells. (TIF) [file ppat.1009241.s006.tif]

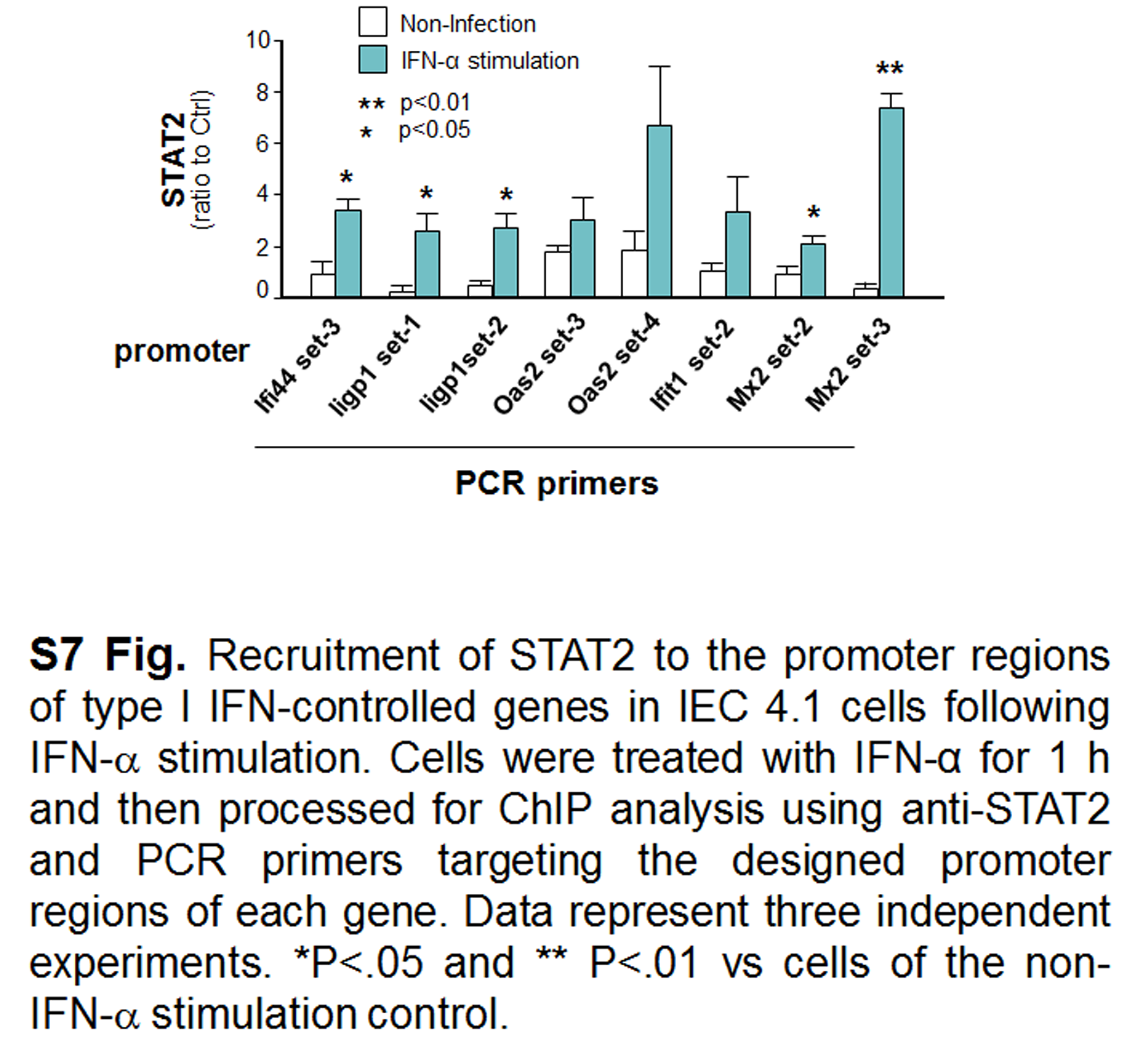

Supplement: S7 Fig — Cells were treated with IFN-α for 1 h and then processed for ChIP analysis using anti-STAT2 and PCR primers targeting the designed promoter regions of each gene. Data represent three independent experiments. *P < .05 and ** P < .01 vs cells of the non-IFN-α stimulation control. (TIF) [file ppat.1009241.s007.tif]

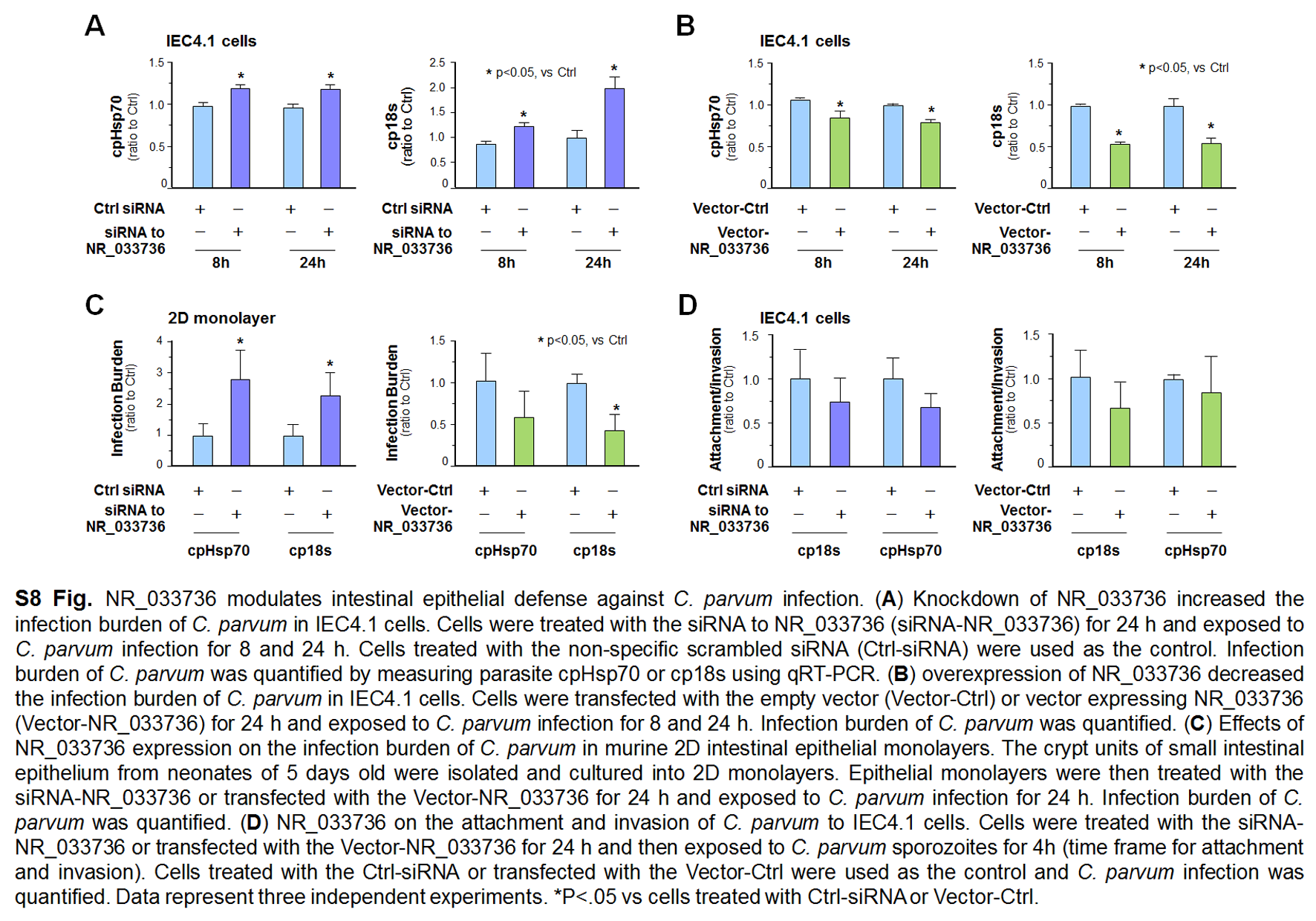

Supplement: S8 Fig — (A) Knockdown of NR_033736 increased the infection burden of C. parvum in IEC4.1 cells. Cells were treated with the siRNA to NR_033736 (siRNA-NR_033736) for 24 h and exposed to C. parvum infection for 8 and 24 h. Cells treated with the non-specific scrambled siRNA (Ctrl-siRNA) were used as the control. Infection burden of C. parvum was quantified by measuring parasite cpHsp70 or cp18s using qRT-PCR. (B) overexpression of NR_033736 decreased the infection burden of C. parvum in IEC4.1 cells. Cells were transfected with the empty vector (Vector-Ctrl) or vector expressing NR_033736 (Vector-NR_033736) for 24 h and exposed to C. parvum infection for 8 and 24 h. Infection burden of C. parvum was quantified. (C) Effects of NR_033736 expression on the infection burden of C. parvum in murine 2D intestinal epithelial monolayers. The crypt units of small intestinal epithelium from neonates of 5 days old were isolated and cultured into 2D monolayers. Epithelial monolayers were then treated with the siRNA-NR_033736 or transfected with the Vector-NR_033736 for 24 h and exposed to C. parvum infection for 24 h. Infection burden of C. parvum was quantified. (D) NR_033736 on the attachment and invasion of C. parvum to IEC4.1 cells. Cells were treated with the siRNA-NR_033736 or transfected with the Vector-NR_033736 for 24 h and then exposed to C. parvum sporozoites for 4h (time frame for attachment and invasion). Cells treated with the Ctrl-siRNA or transfected with the Vector-Ctrl were used as the control and C. parvum infection was quantified. Data represent three independent experiments. *P < .05 vs cells treated with Ctrl-siRNA or Vector-Ctrl. (TIF) [file ppat.1009241.s008.tif]

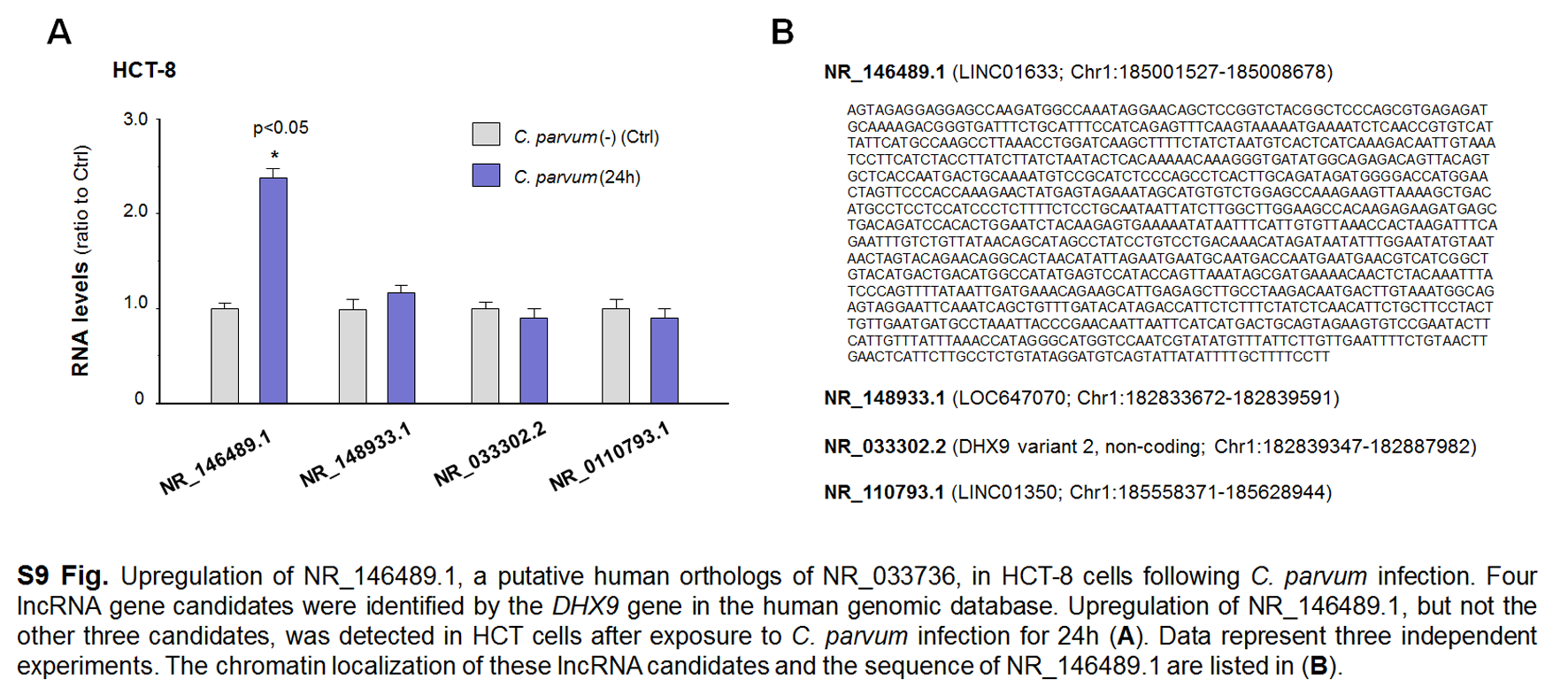

Supplement: S9 Fig — Four lncRNA gene candidates were identified by the DHX9 gene in the human genomic database. Upregulation of NR_146489.1, but not the other three candidates, was detected in HCT cells after exposure to C. parvum infection for 24h (A). Data represent three independent experiments. The chromatin localization of these lncRNA candidates and the sequence of NR_146489.1 are listed in (B). (TIF) [file ppat.1009241.s009.tif]
